# Integrated Transcriptomics and Metabolomics Analyses of Stress-Induced Murine Hair Follicle Growth Inhibition

**DOI:** 10.3389/fmolb.2022.781619

**Published:** 2022-02-07

**Authors:** Xuewen Wang, Changqing Cai, Qichang Liang, Meng Xia, Lihua Lai, Xia Wu, Xiaoyun Jiang, Hao Cheng, Yinjing Song, Qiang Zhou

**Affiliations:** ^1^ Department of Dermatology and Venereology, Sir Run Run Shaw Hospital, Zhejiang University School of Medicine, Hangzhou, China; ^2^ Hair Research Center, Sir Run Run Shaw Hospital, Zhejiang University School of Medicine, Hangzhou, China; ^3^ Yonghe Medical Group Co. Ltd., Beijing, China; ^4^ Institute of Immunology, Zhejiang University School of Medicine, Hangzhou, China

**Keywords:** metabolomics, transcriptomics, hair growth inhibition, chronic restraint stress, hexokinase-1

## Abstract

Psychological stress plays an important role in hair loss, but the underlying mechanisms are not well-understood, and the effective therapies available to regrow hair are rare. In this study, we established a chronic restraint stress (CRS)-induced hair growth inhibition mouse model and performed a comprehensive analysis of metabolomics and transcriptomics. Metabolomics data analysis showed that the primary and secondary metabolic pathways, such as carbohydrate metabolism, amino acid metabolism, and lipid metabolism were significantly altered in skin tissue of CRS group. Transcriptomics analysis also showed significant changes of genes expression profiles involved in regulation of metabolic processes including arachidonic acid metabolism, glutathione metabolism, glycolysis gluconeogenesis, nicotinate and nicotinamide metabolism, purine metabolism, retinol metabolism and cholesterol metabolism. Furthermore, RNA-Seq analyses also found that numerous genes associated with metabolism were significantly changed, such as Hk-1, in CRS-induced hair growth inhibition. Overall, our study supplied new insights into the hair growth inhibition induced by CRS from the perspective of integrated metabolomics and transcriptomics analyses.

## Introduction

As one of the most common skin diseases, hair loss has negative effects on patient’s psychological well-being and reduces their life quality (Williamson et al., 2001). Previous studies indicate that psychoemotional stress plays a pivotal role in triggering and aggravating hair loss, such as alopecia areata (AA), telogen effluvium and androgenetic alopecia (Hadshiew et al., 2004; Peters et al., 2006; Alexopoulos and Chrousos, 2016; Dainichi and Kabashima, 2017). Numerous studies reveal that hair loss is highly related to hair follicle (HF) pathophysiological changes (Cotsarelis and Millar, 2001; Pratt et al., 2017). HFs go through successive cycles of anagen (growth), catagen (regression), and telogen (rest) phases (Millar, 2002; Rishikaysh et al., 2014). The hair follicle cycling is modulated by various signals which control quiescence and activation of hair follicle stem cells (HFSCs) (Chai et al., 2019; Feng et al., 2020).

Psychological stress has been reported to alter the hair cycle via neuroendocrine or neuroimmunological signaling pathways (Paus et al., 2008; Ito, 2010; Paus et al., 2014). The generation of HFs can be affected by numerous neuromediators which regulate HF growth, pigmentation, remodeling, immune status, stem cell biology, and energy metabolism (Peters et al., 2006; Paus et al., 2014; Choi et al., 2021). Numerous studies demonstrate that stress increases apoptotic cells, inhibits hair bulge stem cells and hair bulb keratinocytes proliferation, promotes mast cell degranulation, and induces premature catagen and neurogenic inflammation. In addition, it has been reported that chronic restraint stress (CRS) induces the delay of hair cycle via autophagy (L. Wang et al., 2015). But other researchers find that supplementation of a metabolite a-ketobutyrate (a-KB) in old mice can increase longevity and prevent alopecia by inducing autophagy (Chai et al., 2019). Thus, the mechanisms of CRS on hair growth remains to be further investigated.

Metabolomics is an omics category focused on simultaneous qualitative and quantitative analyses of low molecular-weight metabolites within an organism or cell during a specific physiological period (Christodoulou et al., 2020; Huang et al., 2021). Changes in metabolites play a critical role in various diseases, including hair loss. Clinical investigations have suggested that androgenic alopecia (AGA) patients showed significant abnormal lipid profiles (Antoni and Dhabhar, 2019; Kim et al., 2017). Lipid-modulatory therapies have been reported to alter hair growth (Lattouf et al., 2015; Cervantes et al., 2018; Shin et al., 2021). Moreover, cholesterol is involved in proliferation and differentiation of HF cell population. It is reported that primary cicatricial alopecia is also related to cholesterol metabolism disorders (Palmer et al., 2020).

Intriguingly, psychological stress can trigger metabolic changes and psychological stress is also associated with many metabolic-related diseases including diabetes, cardiovascular disease, as well as cancers (Gu et al., 2012; Hackett and Steptoe, 2017; Antoni and Dhabhar, 2019). CRS also drastically increases the expression of genes related to fatty acid/lipid/sterol metabolism in the liver of mice (Ha et al., 2003). Some researchers also report that HFSCs maintain a dormant metabolic state and could utilize glycolytic metabolism, thus producing more lactate than other cells in the epidermis (Flores et al., 2017)*.* Small molecules that activate autophagy could initiate anagen and stimulate hair growth, including some metabolites associated with carbohydrate metabolism, a-ketoglutarate (a-KG), and a-ketobutyrate (a-KB) (Chai et al., 2019). Obesity-induced stress, such as that induced by a high-fat diet accelerates hair loss mainly through depletion of HFSCs, which indicates metabolic changes may affect hair growth via stem cell inflammatory signals (Morinaga et al., 2021). However, the metabolic pathways and molecules involved in the mechanisms of psychological stress effects on hair growth are still unclear.

Therefore, in order to elucidate the pathogenesis and explore potential therapeutic strategies, our study investigated important biological metabolites, genes and signaling pathways that were related to CRS-induced hair growth inhibition. Our results not only provided a validated and comprehensive understanding of integrated transcriptomics and metabolism analyses in CRS-induced hair growth inhibition but also found some genes including Hk-1 which might be new targets for the treatment of CRS-induced hair growth inhibition.

## Materials and Methods

### Mice

All experiments were approved by the center of experiment animal, Zhejiang University (China). C57BL/6 male mice were obtained at 6–8 weeks of age from Shanghai SLAC Laboratory Animal Co., Ltd. All mice were acclimated for 7 days before the onset of studies at Experimental Animal Center of Zhejiang University (China). The standard conditions of animal facility were maintained as following: temperature 21–24°C; 12 h light/dark cycle (lights on 06:00–18:00); humidity 50–60%. Sterilized water and food were provided ad libitum during this period. The study was approved by the Ethics Committee of Sir Run Run Shaw Hospital of Zhejiang University School of Medicine (Approval no. SRRSH2021401).

### Stress Application and Anagen Induction

The procedure of CRS was conducted as previously reported method and lasted for 20 days (Q. Wang et al., 2019). Mice were placed into 50 ml conical tubes, without physically compressed for 6 h (10:00–16:00) each day (Liu et al., 2013; Zhao et al., 2013). During the period of stress application, control mice were kept undisturbed in their original cages, and all groups of mice were not provided with food and water. On day 8 of the experiment, wax/rosin mixture (1:1 on weight) was applied to the dorsal skin (from neck to tail) of mice to induce anagen. Then we peeled off the mixture and removed all hair shafts to induce synchronization of hair cycle, as evidenced by the homogeneously pink skin color in the back, which indicated all hair follicles in telogen (Müller-Röver et al., 2001). Mice were not exposed to CRS on the day of depilation.

### Assessment of Hair Cycle

Assessment of Hair cycle were based on the appearance of skin pigmentation and hair shaft which were monitored by pictures, as previously described (Stenn and Paus, 2001). To quantify the stage of the hair follicles, skin pigmentation score values from 0 to 100 were calculated based on skin pigmentation levels and hair shaft density, with 0 indicating no hair growth (and no pigmentation) and a higher number corresponding to darker skin and larger areas of dense hair growth (Chai et al., 2019). Briefly, skin pigmentation scored 50 refers to 50 percent of full-length hair shaft on back skin or 100 percent of skin pigmentation without visible hair growth. Skin pigmentation scored 70 refers to 70 percent of full-length hair shaft in back skin or 100 percent of skin pigmentation with 40 percent of full-length hair shaft. Skin pigmentation scored 100 refers to 100 percent of full-length hair shaft on back skin (Feng et al., 2020).

### Tissue Preparation and Immunohistochemistry Staining

C57BL/6J mouse dorsal skin specimens were harvested about 2 × 4 cm on day 21 of the experiment before being collected for histological and molecular analyses. Full-thickness skin tissues (measured thickness 400–700 um) were then fixed in 4% formalin and dehydrated for embedding in paraffin. 5 mm paraffin sections were subjected to hematoxylin and eosin (H&E) staining and immunohistochemistry. The Ki67 (ab15580) antibody was purchased from Abcam. Images were captured using an Olympus microscope (IX73) at X40 and X400 magnification. The remaining skin specimens were immediately snap frozen in liquid nitrogen and stored at −80°C for subsequent use.

### Library Construction, RNA Sequencing and Primary Analysis

Three replicate samples of control and CRS C57BL/6 mice dorsal skin specimens were used for library construction and RNA sequencing, respectively. Total RNA was isolated from skin tissues and purified using TRIzol reagent (Invitrogen, Carlsbad, CA, United States) following the manufacturer’s procedure. The NanoDrop ND-1000 (NanoDrop, Wilmington, DE, United States) was used to quantify the amount of RNA and purity of each sample. The Bioanalyzer 2,100 (Agilent, CA, United States) with RIN number >7.0 was used to assess the integrity of RNA, which was also confirmed by electrophoresis with denaturing agarose gel. Poly (A) RNA was purified from 1 μg total RNA by Dynabeads Oligo (dT)25–61,005 (Thermo Fisher, CA, United States) and was fragmented into small pieces using Magnesium RNA Fragmentation Module (NEB, cat. e6150, United States) under 94°C 5–7 min. The SuperScript™ II Reverse Transcriptase (Invitrogen, cat. 1896649, United States) was used to reverse-transcribe the cleaved RNA fragments to create the cDNA, which were then transform to the U-labeled second-stranded DNAs with E. coli DNA polymerase I (NEB, cat. m0209, United States), RNase H (NEB, cat. m0297, United States) and dUTP Solution (Thermo Fisher, cat. R0133, United States). An A-base was then added to the blunt ends of each strand, preparing them for ligation to the sequencing adapters. Subsequently, the ligated products were amplified with PCR amplification. At last, the Illumina Novaseq™ 6,000 (LC-Bio Technology CO., Ltd., Hangzhou, China) was used to perform the 2 × 150bp paired-end sequencing (PE150). The differentially expressed mRNAs were selected with fold change >2 or fold change <0.5 and *p* value <0.05 by R package edgeR (https://bioconductor.org/packages/release/bioc/html/edgeR.html).

### Metabolite Extraction and LC-MS Analysis

Metabolomics sample collection, preparation, and metabolome profiling were carried out as previously described (Ruiying et al., 2020). The back skin tissues from mice treated with CRS or control were thawed on ice, and metabolites were extracted from 20 µL of each sample using 120 µL of precooled 50% methanol buffer (methanol and distilled water were mixed in a 1:1 ratio). Then the mixture of metabolites was vortexed for 1 min and incubated for 10 min at room temperature, and stored at −20°C overnight. The mixture was centrifugated at 4,000 g for 20 min, subsequently the supernatant was transferred to 96-well plates. The samples were stored at −80 °C prior to the LC-MS analysis. Pooled quality control (QC) samples were also prepared by combining 10 μL of each extraction mixture. All samples were detected by a Triple TOF 5600 Plus high-resolution tandem mass spectrometer (SCIEX, Warrington, United Kingdom) with both positive and negative ion modes. Chromatographic separation was performed using an ultraperformance liquid chromatography (UPLC) system (SCIEX, United Kingdom). The data acquisition mode was DDA.

### Data Processing and Annotation

The XCMS software was used to acquire the LC-MS pretreatment data including peak picking, peak grouping, retention time correction, second peak grouping, and annotation of isotopes and adducts. Raw data files were transformed into mzXML format and then processed by the XCMS, CAMERA and metaX toolbox included in R software. The comprehensive information of retention time and m/z data was identified for each ion, recorded the intensity of each peak, generated a three-dimensional matrix containing arbitrarily assigned peak indices (retention time-m/z pairs), sample names (observations) and ion intensity information (variables), and matched to the in-house and public database. The metabolites by matching the exact molecular mass data (m/z) to those from the database within a threshold of 10 ppm was annotated by the open access databases, Kyoto Encyclopedia of Genes and Genomes (KEGG) and Human Metabolome Database (HMDB). The metaX was used to further preprocess the peak intensity data. Those features that were detected less than 50 percent of QC samples or 80 percent of test samples were removed, and values for missing peaks were imputed with the k-nearest neighbor algorithm to improve the quality of data. Principal component analysis (PCA) was used to identify outliers and batch effects using the pre-processed dataset. To minimize signal intensity drift over time, QC-based robust LOESS signal correction was used to fit to the QC data. Besides, the relevant standard deviations of the metabolic features were calculated across all QC samples, and those with standard deviations >30 percent were removed. All the annotated secondary metabolites and their Metabolomics Standard Initiative (MSI) level are showed in Supplementary Table S1.

The group datasets were normalized before analysis was performed. Data normalization was carried out using the probabilistic quotient normalization algorithm. Differential enrichment of metabolite features between CRS and control groups was analyzed by Student’s t-test FDR-adjusted *p*-value less than 0.05. Then, QC-robust spline batch correction was performed using QC samples. Supervised partial least-squares discriminant analysis (PLS-DA) was conducted through metaX to discriminate the different variables between the groups. The Variable Important for the Projection (VIP) cut-off value of 1.0 was set to select important features.

### Joint Analysis of Metabolites and Genes

Metabolites and genes in the same pathways were always dysregulated together, so we utilized a pathway-based approach and integrated different levels of omics in the biological process. Enriched differential genes and metabolites were used in the joint pathway module for integrative analysis in MetaboAnalyst5.0. After uploaded our differential metabolites on MetaboAnalyst (https://www.metaboanalyst.ca/), the metabolites were then mapped to KEGG metabolic pathways for enrichment analysis.

### Statistical Analyses

The statistical analyses were expressed as the mean ± SD and performed using GraphPad Prism software (v.8.0). Statistical significance between two groups was determined by Student’s t-test. All experiments are repeated three times independently. Asterisk coding is indicated in Figure legends as **, *p* < 0.01.

## Results

### CRS Significantly Suppresses Hair Growth

**FIGURE 1:**
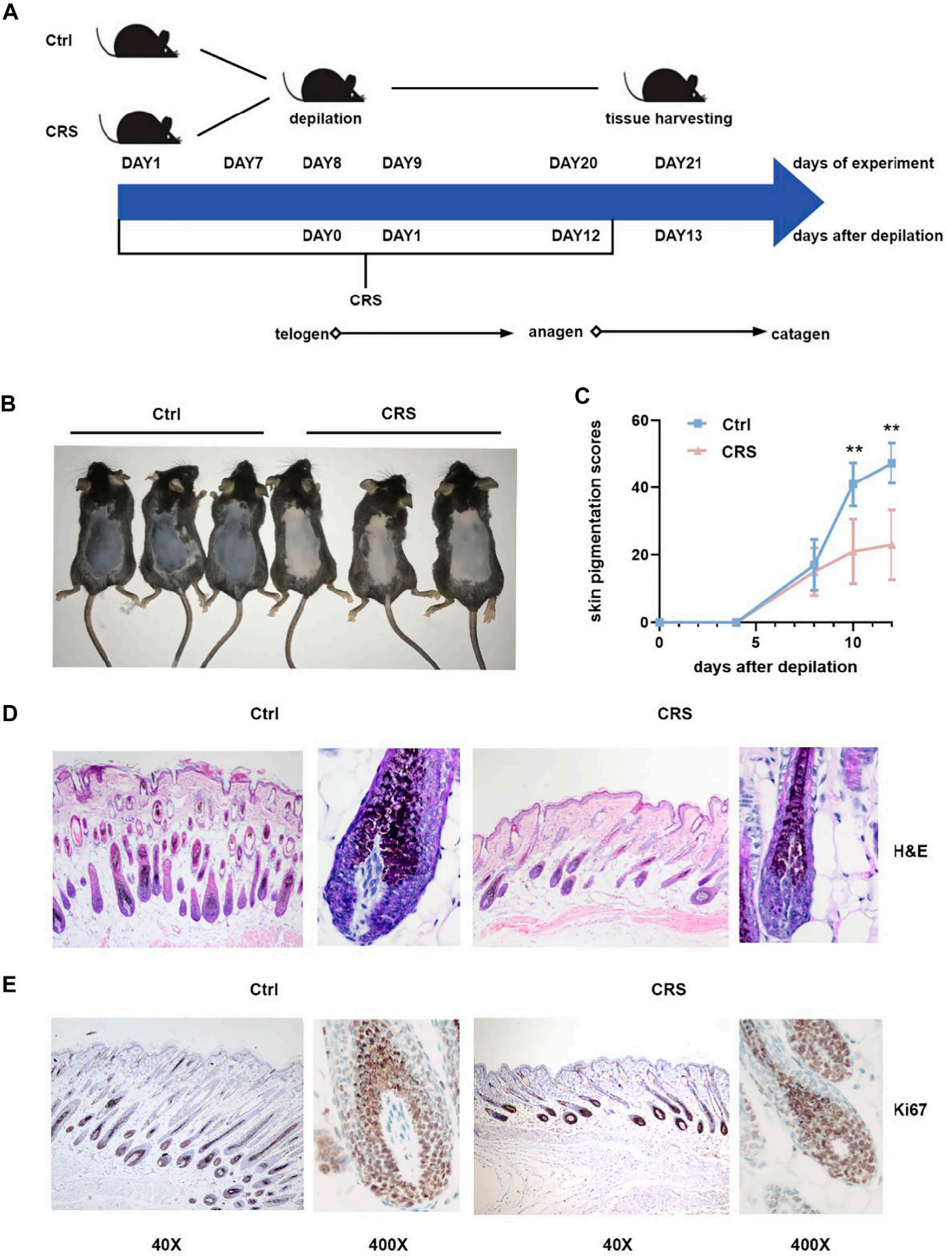
CRS significantly suppresses hair growth. **(A)** Flow chart of the animal experiment. The CRS treatment was applied from day1 and lasted for 20 days. The depilation of mice was on day 8 of the experiment, without applying CRS. On day 21 of the experiment, all animals were sacrificed for histological and metabolomics and transcriptomics analysis. **(B)** Photograph shown was taken on day 21 of experiment in (A), by which time mice treated with CRS exhibited hair growth inhibition versus control. **(C)** Skin melanin pigmentation scores (described in Materials and Methods) of murine dorsal skins treated with CRS versus control. Data are represented as mean ± SD. n = 5 mice in each group. *p* values are determined by Student’s t-test. **p < 0.01 compared with control group. **(D)** H&E staining showed the morphological changes in hair follicle. Magnification: 40×on the left; 400× on the right. **(E)** Immunohistochemistry for Ki-67 in back skin sections obtained from CRS group and control group. Magnification: 40× on the left; 400× on the right.

To confirm the inhibition of hair growth induced by CRS, we established the inhibition of hair growth affected by CRS model on C57BL/6 mice (Figure 1A). The dorsal skin color of the mice was pink in the telogen phase on the day of depilation and gradually became black, as the melanogenic activity of follicular melanocytes is related to the anagen stage of the hair cycle (Q. Wang et al., 2019). As shown in Figure 1B, on day 12 after depilation, no pigmentation or only a few scattered pigmented spots were visible on the dorsal skin of mice in the CRS group. In contrast, skin pigmentation was apparent in the control group, and some of the hair shafts were visible (Figure 1B). Statistical analyses also showed the skin pigmentation scores of murine dorsal skins in CRS group are significantly less than the control group on 10 days after depilation (day 18 of experiment) (*p <* 0.01) (Figure 1C).

Next, we took advantage of H&E staining and immunohistochemistry to detect the formation and proliferation of hair follicles. Compared to controls, CRS dramatically decreased the number of hair follicles, the length of hair shafts, and the thickness of dermal layers (Figure 1D). The expression of proliferation marker Ki-67 (Magerl et al., 2001) was lower in hair follicles of the CRS group than that of control group (Figure 1E). Our results demonstrated that CRS significantly suppressed the hair growth of dorsal skin in mice.

### CRS Significantly Regulates Metabolic Profile of the Skin Tissue

To systematically analyze the metabolic changes affected by CRS in hair growth, we performed the metabolomic analysis of dorsal skin between CRS group and control group. Compared to the control group, 158 features were significantly down-regulated and 138 features were significantly up-regulated in skin tissues of CRS group (Supplementary Figure S1A). PCA based on metabolite analysis showed that skin tissues of CRS-treated group were distinct from control group (Supplementary Figure S1B). PLS-DA was used to supervise the data analysis, and the permutation test was used to prevent PLS-DA model overfitting (Supplementary Figure S1C). In this study, the CRS group and control group were easily distinguished and the PLS-DA model was reliable (Supplementary Figure S1D). The aligned total ion chromatograms (TICs) and retention time width of all the groups in negative mode were shown in Supplementary Figure S2A, and those in positive modes were shown in Supplementary Figure S2B. Analysis of other checking parameters, including average m/z distribution, metabolite intensity distribution and coefficient of variation distribution, indicated effective sample preparation and high-quality raw data (Supplementary Figures S3A,B).

As shown in Supplementary Figure S4, we identified the primary metabolites with positive and negative ion modes by HMDB database. Among these features, the largest group was “lipids and lipid-like molecules”. The amino acids and the carbohydrates that we identified were belong to “Organic acids and derivatives” HMDB super class and “Organic oxygen compounds” HMDB super class, respectively. To facilitate the observation of metabolic changes, we normalized significantly differential metabolites and created a heatmap of all the secondary metabolites (Supplementary Figure S5).

### CRS Significantly Affects the Profiles of Primary Metabolites in Skin Tissues

**FIGURE 2:**
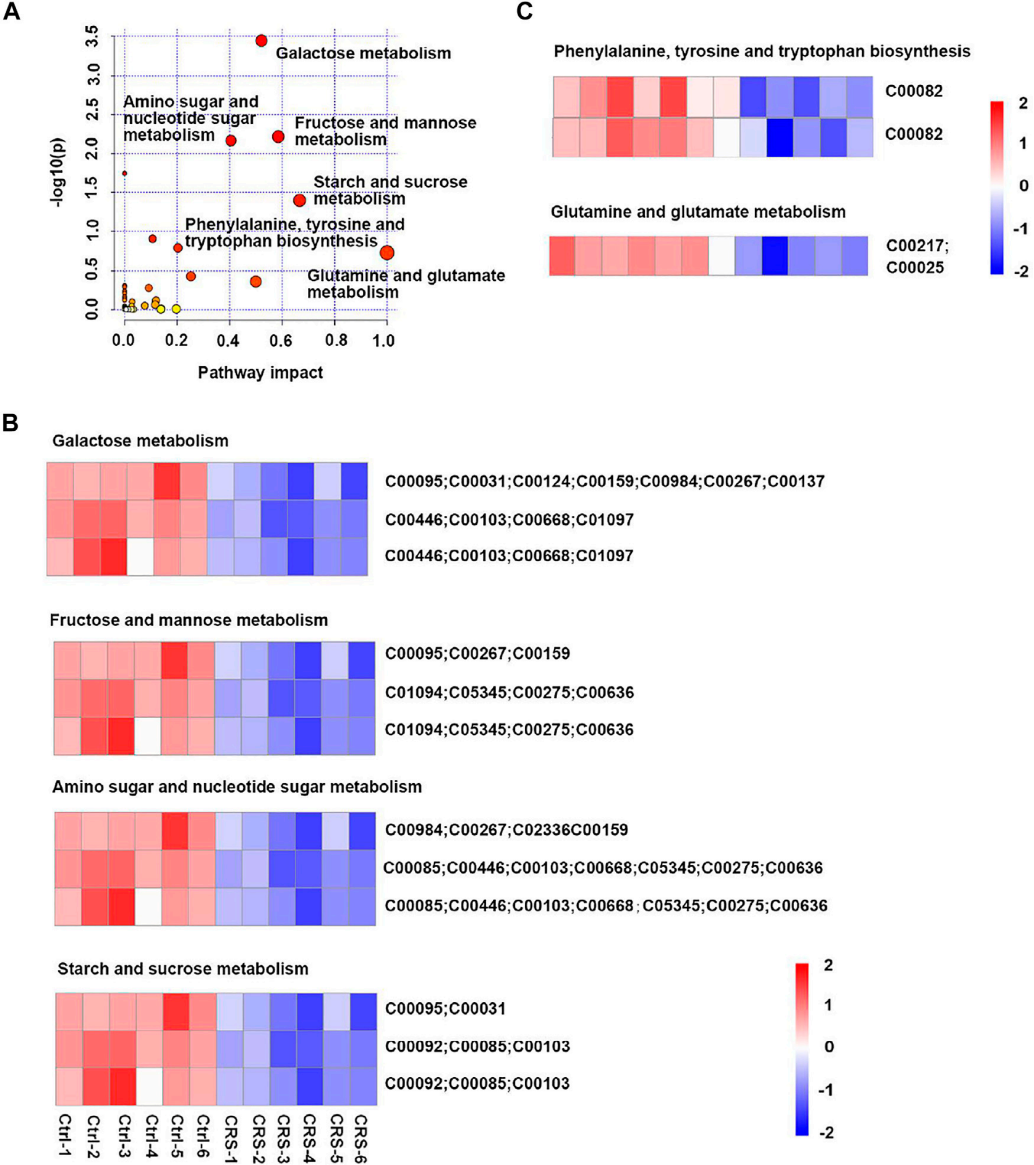
CRS significantly affects the profiles of primary metabolites in skin tissues. **(A)** Scatterplot of enriched KEGG pathways in primary metabolites when comparing CRS group with control group using the MetaboAnalyst 5.0 pathway analysis module. Color shift indicates level of significance, size of dots correlates with the number of differential metabolites. The darker the color and the larger the dot, the stronger is the significance. Top enriched metabolic pathways were labeled. **(B)** Heatmap analysis showed differential metabolites in galactose metabolism (C00095, C00031, C00124, C00159, C00984, C00267, C00137, C00446, C00103, C00668, and C01097), fructose and mannose metabolism (C00095, C00267, C00159, C01094, C05345, C00275, and C00636), amino sugar and nucleotide sugar metabolism (C00984, C00267, C02336, C00159, C00085, C00446, C00103, C00668, C05345, C00275, and C00636), starch and sucrose metabolism (C00095, C00031, C00092, C00085, and C00103) between CRS group and control group. The ordinate was the KEGG ID matched to database. **(C)** Heatmap analysis showed differential metabolites in phenylalanine, tyrosine and tryptophan biosynthesis (C00082), glutamine and glutamate metabolism (C00217, C00025) pathways between CRS group and control group. The ordinate was the KEGG ID matched to database. The color blocks represented the relative expression of metabolites, red represented up-regulation, and blue represented down-regulation.

Metabolic pathways identified by MetaboAnalyst 5.0 for primary metabolites differentially identified by positive and negative polarity ionization in the skin tissue of CRS-treated mice compared to those in control mice are shown in Figure 2A. Among the relevant pathways identified, galactose metabolism (C00095, C00031, C00124, C00159, C00984, C00267, C00137, C00446, C00103, C00668, andC01097), fructose and mannose metabolism (C00095, C00267, C00159, C01094, C05345, C00275, and C00636), amino sugar and nucleotide sugar metabolism (C00984, C00267, C02336, C00159, C00085, C00446, C00103, C00668, C05345, C00275, and C00636), starch and sucrose metabolism (C00095, C00031, C00092, C00085, C00103), phenylalanine, tyrosine and tryptophan biosynthesis (C00082), glutamine and glutamate metabolism (C00217, C00025) were found to be the most important significant metabolic pathways (Figures 2B,C). These pathways were mainly involved in carbohydrate metabolism (fructose and mannose metabolism, galactose metabolism, amino sugar and nucleotide sugar metabolism, starch and sucrose metabolism) (Figure 2B) and amino acid metabolism (phenylalanine, tyrosine and tryptophan biosynthesis, glutamine and glutamate metabolism) (Figure 2C). It has been reported glutamine and glutamate metabolism play important roles in the epidermis and stem cells metabolism (C. S. Kim et al., 2020; Simsek et al., 2010; Takubo et al., 2013). Details of differential metabolites in skin tissues are shown in Supplementary Table S2.

### CRS Significantly Affects the Profiles of Secondary Metabolites in Skin Tissues

**FIGURE 3:**
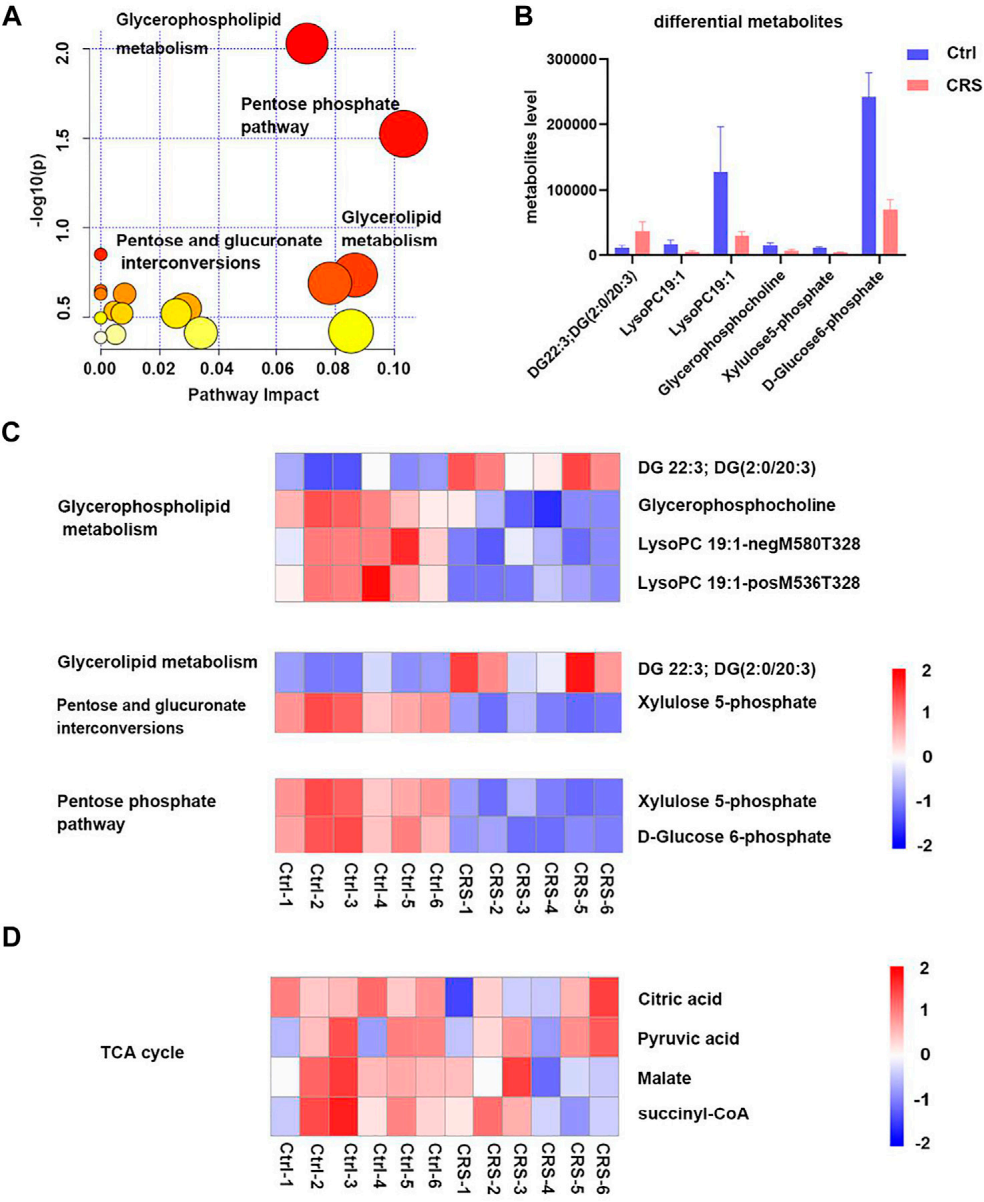
CRS significantly affects the profiles of secondary metabolites in skin tissues. **(A)** Scatterplot of enriched KEGG pathways in secondary metabolites when comparing CRS group with control group using the MetaboAnalyst 5.0 pathway analysis module. Top enriched metabolic pathways were labeled. **(B)** Comparison of secondary metabolites level between control group and CRS group in the top 4 significant pathways. **(C)** Heatmap analysis showed differential metabolites in glycerophospholipid metabolism, pentose phosphate pathway, glycerolipid metabolism, pentose and glucuronate interconversions pathways. **(D)** Heatmap analysis showed metabolites changes in TCA cycle.

The significant differential secondary metabolites were subjected for KEGG pathway analysis. As shown in Figure 3A, the most significantly changed pathway was the glycerophospholipid metabolism. Pentose phosphate pathway, glycerolipid metabolism, pentose and glucuronate interconversions were also significantly altered after CRS treatment. A total of six significant differential secondary metabolites were identified, including DG 22:3; DG (2:0/20:3) which was significantly upregulated, LysoPC 19:1-neg-M580T328, LysoPC 19:1-pos-M536T328, glycerophosphocholine, xylulose 5-phosphate and D-Glucose 6-phosphate which were significantly downregulated (Figures 3B,C). Among them, D-Glucose 6-phosphate was the most drastically reduced metabolite. In addition, D-Fructose 6-phosphate was also significantly downregulated in CRS group in primary metabolites. Both D-Glucose-6-phosphate and D-Fructose 6-phosphate are involved in glycolytic metabolism pathways, which indicated that glycolytic metabolism might play a critical role in the inhibition of hair growth induced by CRS. Conversely, metabolites in TCA cycle were not significantly changed between the CRS group and control group (Figure 3D). Collectively these results suggested that although skin tissue use the TCA cycle to generate energy, CRS could not regulate TCA metabolism to inhibit hair growth.

Furthermore, KEGG pathway enrichment analyses were conducted to further analyze the metabolic profiles in skin tissues of CRS-induced hair growth inhibition. The top 20 KEGG pathways were shown in Supplementary Figure S6. These results demonstrated that biosynthesis of amino acids, central carbon metabolism in cancer, protein digestion and absorption, ABC transporters, glycerophospholipid metabolism, carbon metabolism pathways were the most significantly altered pathways.

### CRS Significantly Affects Genes Expression Associated With Primary Metabolites

**FIGURE 4:**
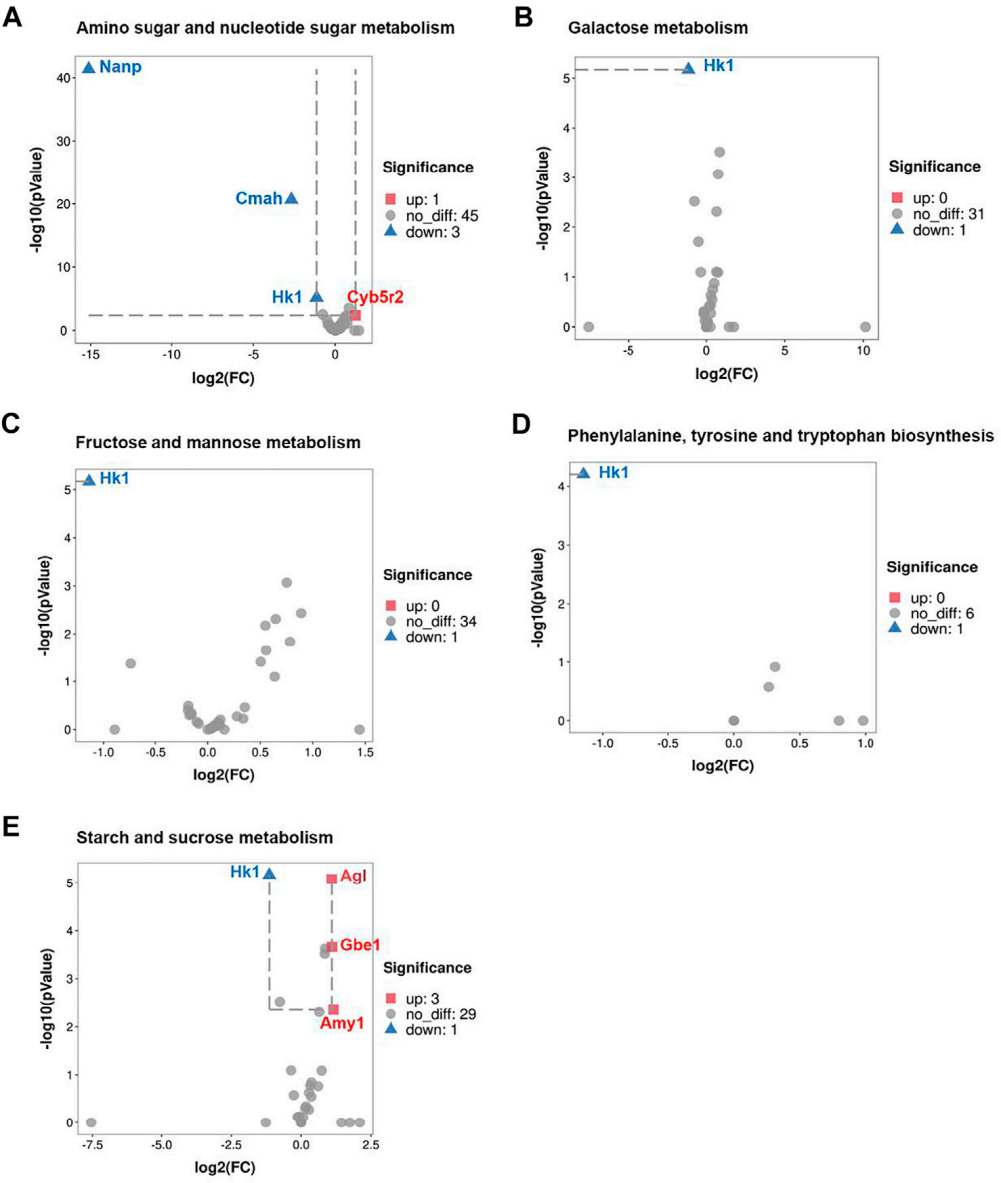
CRS significantly affects genes expression associated with primary metabolites. **(A)** Volcano plot showed regulation of genes expression in amino sugar and nucleotide sugar metabolism. Significantly DEGs were labeled. FC is for gene expression fold change in CRS group compared to control group. The DEGs were selected with fold change >2 or fold change <0.5 and *p* value <0.05. **(B)** Volcano plot showed regulation of genes expression in galactose metabolism. **(C)** Volcano plot showed regulation of genes expression in fructose and mannose metabolism. **(D)** Volcano plot showed regulation of genes expression in phenylalanine, tyrosine and tryptophan biosynthesis. **(E)** Volcano plot showed regulation of genes expression in starch and sucrose metabolism.

Among these pathways above, we identified several differentially expressed genes (DEGs) based on KEGG pathway analysis in transcriptomic. Volcano plot analysis indicated that a total of 5 pathways were matched to significantly DEGs for primary metabolites, including galactose metabolism, fructose and mannose metabolism, amino sugar and nucleotide sugar metabolism, phenylalanine, tyrosine and tryptophan biosynthesis, starch and sucrose metabolism. As shown in Figure 4, Hk-1 was significantly downregulated in all 5 metabolic pathways, suggested that Hk-1 played a very important role in relative biological process of CRS inhibiting hair growth. Furthermore, some other genes expression was also significantly changed. In amino sugar and nucleotide sugar metabolism, *Nanp,* and *Cmah* expression were significantly down-regulated and *Cyb5r2* was significantly up-regulated (Figure 4A). The *Nanp* gene is related to synthesis of substrates in N-glycan biosynthesis and metabolism of proteins pathways, *Cmah* encodes cytidine monophosphate-N-acetylneuraminic acid hydroxylase, an enzyme responsible for Neu5Gc biosynthesis (Burzyńska et al., 2021), and *Cyb5r2* encodes Cytochrome B5 Reductase 2 which participates in many processes including cholesterol biosynthesis, fatty acid desaturation and elongation. *Agl, Gbe1, and Amy1* were significantly up-regulated which related to starch and sucrose metabolism (Figure 4E). *Agl* encodes the glycogen debrancher enzyme that is involved in glycogen degradation, *Gbe1* encodes the glycogen branching which is important to increase the solubility of the glycogen molecule and, consequently, reducing the osmotic pressure within cells (Malinska et al., 2020). *Amy1* encodes amylase alpha produced by the salivary gland. Amylases catalyze the first step in digestion of dietary starch and glycogen. Previous researches have repeatedly demonstrated the activation of salivary alpha-amylase induced by psychosocial stress (Skosnik et al., 2000; Rohleder et al., 2004), which may explain the up-regulation of *Amy1* in starch and sucrose metabolism after treated with CRS. The FPKM values of differentially expressed genes associated with primary metabolites were shown in Supplementary Table S3.

### CRS Significantly Affects Genes Expression Associated With Secondary Metabolites

**FIGURE 5:**
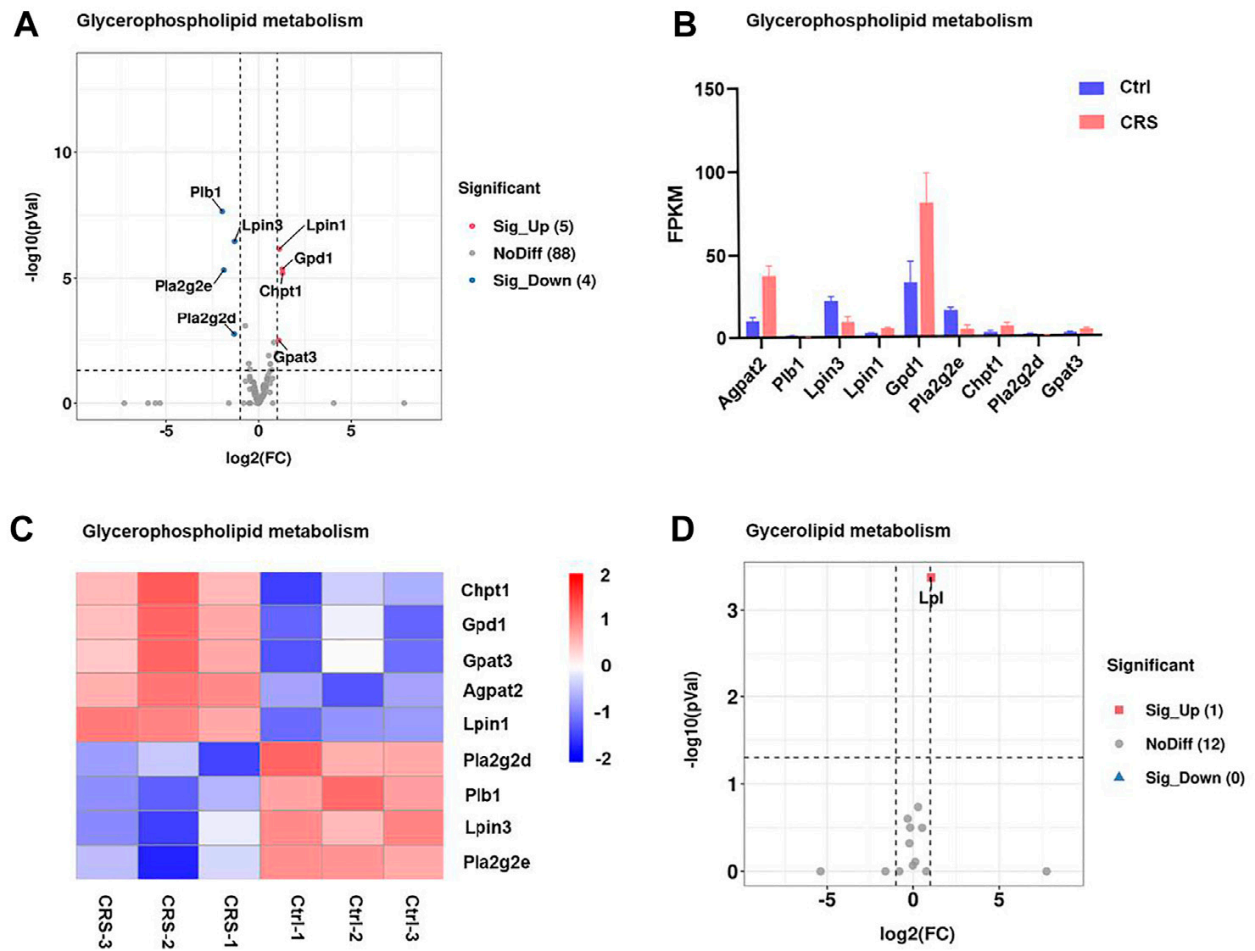
CRS significantly affects genes expression associated with secondary metabolites. **(A)** Volcano plot showed regulation of genes expression in glycerophospholipid metabolism. Significantly DEGs were labeled. FC is for gene expression fold change in CRS group compared to control group. The DEGs were selected with fold change >2 or fold change <0.5 and *p* value <0.05. **(B)** The fragments per kilobase of transcript per million mapped reads (FPKM) values of DEGs in glycerophospholipid metabolism. **(C)** Heatmap analysis showed regulation of genes expression in glycerophospholipid metabolism. The color blocks represented the relative genes expression, red represented up-regulation, and blue represented down-regulation. Volcano plot showed regulation of gene expression in glycerolipid metabolism.

Consistent with the metabolomics analysis for secondary metabolites, the expression of genes associated with glycerophospholipid metabolism and glycerolipid metabolism pathways were also significantly changed. Among a total of 97 genes involved in the pathway of glycerophospholipid metabolism, 9 genes (*Agpat2, plb1, Gpd1, Pla2g2e, Pla2g2d, Gapt3, Lpin3, Lpin1, and Chpt1*) were significantly changed (Figure 5A). Among these genes, *Agpat2* and *Gpd1* were markedly up-regulated (Figures 5B,C). *Gpd1* plays a critical role in carbohydrate and lipid metabolism. *Agpat2* converts lysophosphatidic acid to phosphatidic acid, the second step in *de novo* phospholipid biosynthesis. In addition, *Lpl* which encodes lipoprotein lipase was involved in the glycerolipid metabolism, and the gene expression was also drastically increased (Figure 5D). The FPKM values of differentially expressed genes associated with secondary metabolites were shown in Supplementary Table S4. The remarkable regulation of these genes suggested the impact of CRS on hair growth is strongly linked to lipid metabolism.

### CRS Significantly Alters Genes Expression Related to Metabolism Pathways Based on RNA-Seq

To further analyze broad metabolic pathways of CRS participation in hair growth inhibition, KEGG pathway enrichment analyses were conducted and revealed that CRS also affected gene expression in numerous other metabolic pathways, including arachidonic acid metabolism, glutathione metabolism, glycolysis gluconeogenesis, nicotinate and nicotinamide metabolism, purine metabolism, retinol metabolism and ABC transporters. A total of 97 genes associated with metabolism were significantly differentially expressed in the skin of CRS group compared to that of control group (Supplementary Figure S7).

**FIGURE 6:**
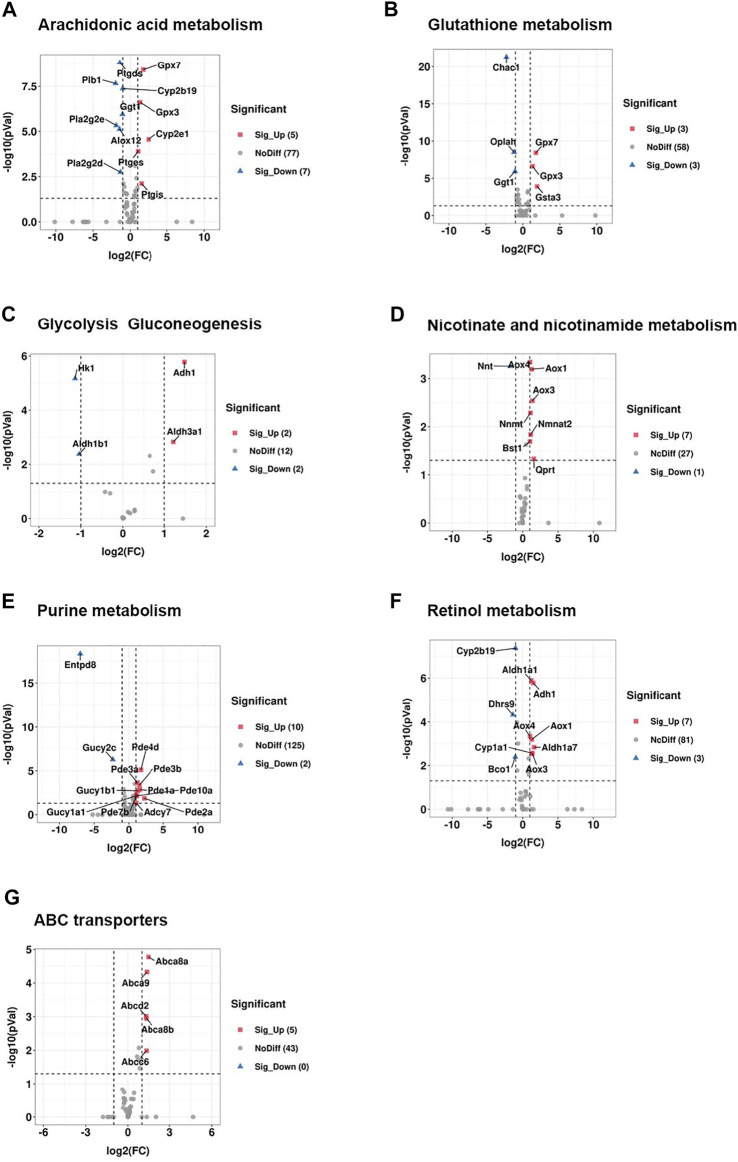
CRS significantly alters genes expression related to metabolism pathways based on RNA-seq. **(A)** Volcano plot showed regulation of genes expression in arachidonic acid metabolism between CRS group and control group. Significantly DEGs were labeled. FC is for gene expression fold change in CRS group compared to control group. The DEGs were selected with fold change >2 or fold change <0.5 and *p* value <0.05. **(B)** Volcano plot showed regulation of genes expression in glutathione metabolism between CRS group and control group. **(C)** Volcano plot showed regulation of genes expression in glycolysis gluconeogenesis between CRS group and control group. **(D)** Volcano plot showed regulation of genes expression in nicotinate and nicotinamide metabolism between CRS group and control group. **(E)** Volcano plot showed regulation of genes expression in purine metabolism between CRS group and control group. **(F)** Volcano plot showed regulation of genes expression in retinol metabolism between CRS group and control group. **(G)** Volcano plot showed regulation of genes expression in ABC transporters between CRS group and control group.

**FIGURE 7:**
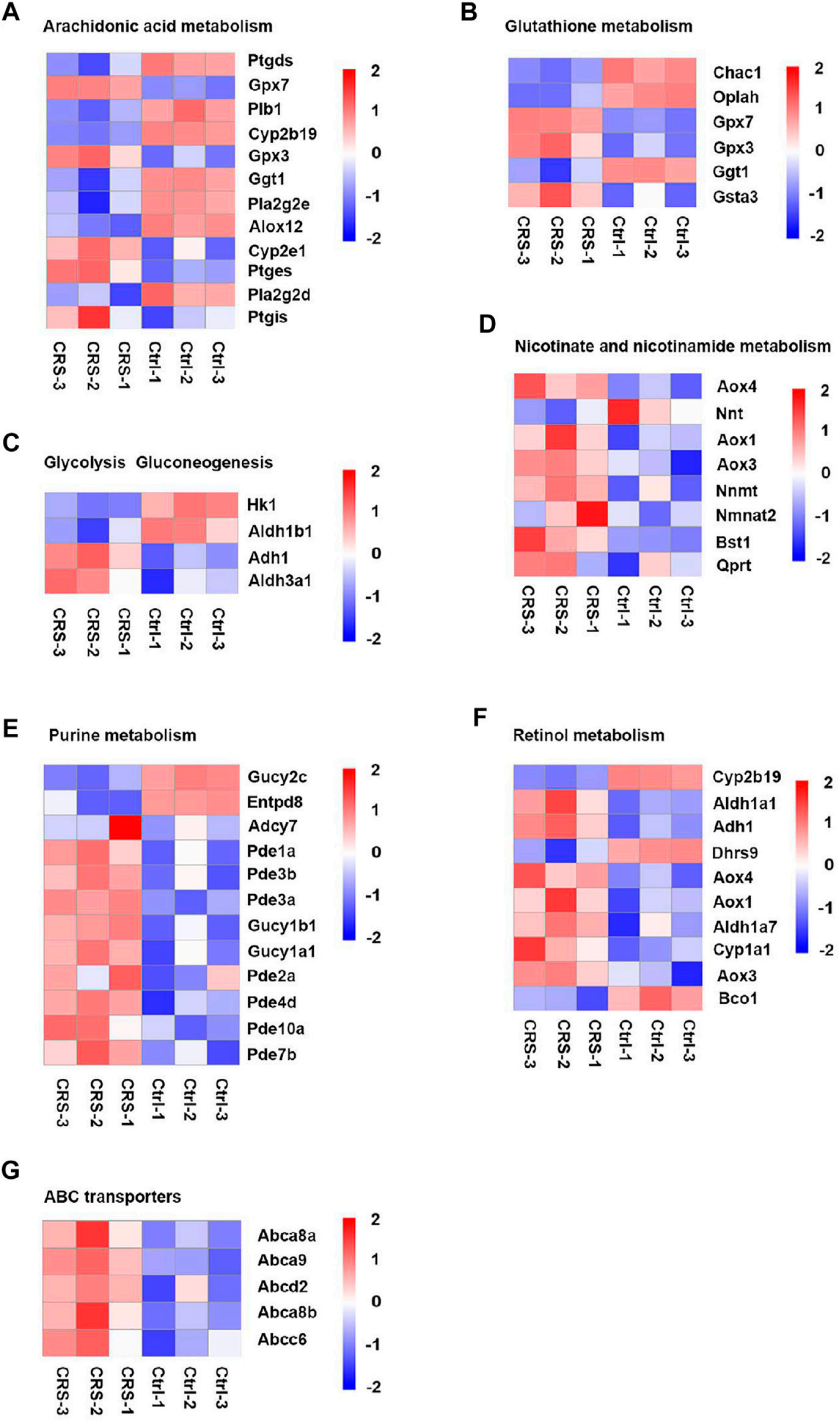
CRS significantly alters genes expression related to metabolism pathways based on RNA-seq. D. Heatmap analysis showed regulation of genes expression in arachidonic acid metabolism between CRS group and control group. **(A)** Heatmap analysis showed regulation of genes expression in glutathione metabolism between CRS group and control group. **(B)** Heatmap analysis showed regulation of genes expression in glycolysis gluconeogenesis between CRS group and control group. **(C)** Heatmap analysis showed regulation of genes expression in nicotinate and nicotinamide metabolism between CRS group and control group. **(D)** Heatmap analysis showed regulation of genes expression in purine metabolism between CRS group and control group. **(E)** Heatmap analysis showed regulation of genes expression in retinol metabolism between CRS group and control group. **(F)** Heatmap analysis showed regulation of genes expression in ABC transporters between CRS group and control group.

**FIGURE 8:**
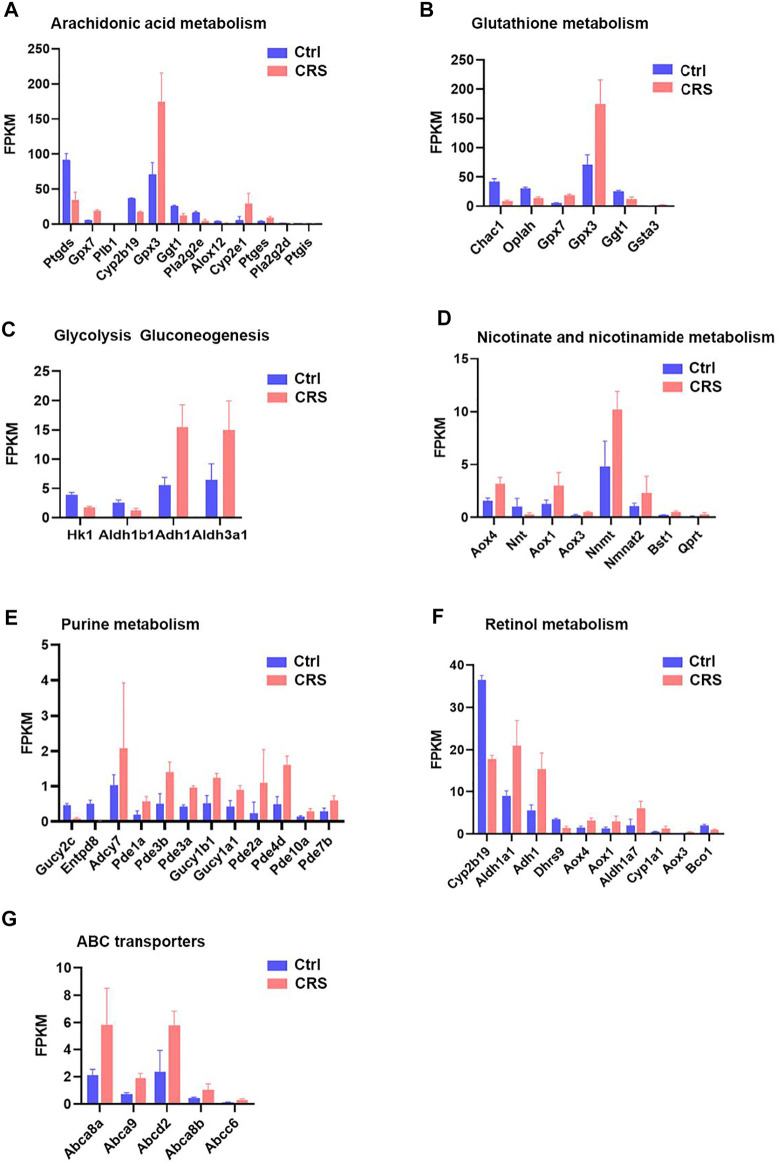
CRS significantly alters genes expression related to metabolism pathways based on RNA-seq. **(A)** The FPKM values of DEGs associated with arachidonic acid metabolism between CRS group and control group. **(B)** The FPKM values of DEGs associated with glutathione metabolism between CRS group and control group. **(C)** The FPKM values of DEGs associated with glycolysis gluconeogenesis between CRS group and control group. **(D)** The FPKM values of DEGs associated with nicotinate and nicotinamide metabolism between CRS group and control group. **(E)** The FPKM values of DEGs associated with purine metabolism between CRS group and control group. **(F)** The FPKM values of DEGs associated with retinol metabolism between CRS group and control group. **(G)** The FPKM values of DEGs associated with ABC transporters between CRS group and control group.

Among a total of 89 genes involved in the pathway of arachidonic acid (AA) metabolism, 12 genes were significantly changed between CRS group and control group, including 5 genes that were upregulated and 7 genes that were downregulated. The upregulated genes included *Gpx7, Gpx3, Cyp2e1, Ptges, Ptgis* while *Ptgds, Plb1, Cyp2b19, Ggt1, Pla2g2e, Pla2g2d, Alox12* were dramatically downregulated (Figures 6A, 7A, 8A). Arachidonic acid is one of the major polyunsaturated fatty acids in mammals (Yu and Wang, 2021). In consideration of the significant decrease of AA-residue-enriched LPCs (e.g., LysoPC 19:1-neg-M580T328, LysoPC 19:1- pos-M536T328etc.), we speculated that arachidonic acid (AA) metabolism was critical for CRS-induced hair growth inhibition.

CRS also affected the expression of genes clustered into glutathione metabolism, including 3 downregulated genes (including *Chac1, Oplah, Ggt1*) and 3 upregulated genes (including *Gpx7, Gpx3, Gsta3*) (Figures 6B, 7B, 8B). For glycolysis gluconeogenesis, CRS significantly suppressed the expression of 2 genes (*Hk1, Aldh1b1*) and increased the expression of 2 genes (*Adh1, Aldh3a1*) (Figures 6C, 7C, 8C). For nicotinate and nicotinamide metabolism, 7 genes exhibited increased expression in CRS group (*Aox4, Aox1, Aox3, Nnmt, Nmnat2, Bst1, Qprt*) and 1 gene exhibited decreased expression (*Nnt*) (Figures 6D, 7D,8D). In addition, there were 12 DEGs between CRS group and control group that clustered to the purine metabolism pathway of which expression was decreased for 2 genes, including *Gucy2c* and *Entpd8*, and increased for 10 genes, including *Adcy7, Pde1a, Pde3b, Pde3a, Gucy1b1, Gucy1a1, Pde2a, Pde4d, Pde10a, Pde7b* (Figures 6E, 7E, 8E). A total of 10 DEGs clustered to the pathway of retinol metabolism after CRS treatment, including 3 genes with decreased expression (*Cyp2b19, Dhrs9* and *Bco1*), and 7 genes with increased expression, including *Aldh1a1, Adh1*, *Aox4, Aox1, Aldh1a7, Cyp1a1, Aox3* (Figures 6F, 7F, 8F)*.* CRS also significantly upregulated the expression of genes important for ABC transporters including *Abca8a, Abca9, Abcd2, Abca8b, Abcc6* (Figures 6G, 7G, 8G)*.* The FPKM values of differentially expressed genes associated with arachidonic acid metabolism, glutathione metabolism, glycolysis gluconeogenesis, nicotinate and nicotinamide metabolism, purine metabolism, retinol metabolism, ABC transporters were shown in Supplementary Tables S5–11. These results showed that the gene expression profiles of multiple metabolic pathways were significantly different between CRS group and control group.

## Discussion

Previous studies have reported that CRS influences hair growth via various hormones, neuropeptides, and neurotransmitters, but little is known about its regulation from the perspective of metabolic signals (Paus et al., 2014). Substance P(SP), Calcitonin gene-related peptide (CGRP) and nerve growth factors (NGF) have been regarded as the critical mediators in stress-induced hair loss (Arck et al., 2005; Samuelov et al., 2012). It has also been demonstrated that the existence of a “brain-hair follicle axis” (BHA), and some neuropeptides such as CGRP, SP and NGF could induce apoptosis of murine follicular keratinocytes and stimulate mast cell degranulation, thus inhibiting hair growth (Arck et al., 2001; Arck, Handjiski, Peters, Hagen, et al., 2003; Arck, Handjiski, Peters, Peter, et al., 2003).

However, psychological stress could also affect metabolic levels, but the underlying molecular mediators are poorly defined (Noerman et al., 2020). Our results suggested that CRS significantly suppressed hair growth and showed significant changes in metabolites between CRS and control group. In this study, we found that some metabolites related to lipid metabolism were significantly changed. Notably, DG 22:3; DG (2:0/20:3) was increased in both glycerophospholipid metabolism and glycerolipid metabolism pathways, while lysophosphatidylcholine (LysoPC 19:1) was decreased robustly. Diacylglycerol (DG), as one of the primary lipid sub-groups in living systems and a second messenger in multiple cell activities, serve as a critical role in hastening the β-oxidation of fatty acids, as well as influence the expression of lipid metabolism-linked genes (Almena and Mérida, 2011; Eichmann and Lass, 2015). Importantly, it has been shown that chronic stress alters the levels of DG in stress-susceptible brain regions (Patel et al., 2009; Oliveira et al., 2016). The high level of DG, such as DG 22:3; DG (2:0/20:3), in the dorsal skin of CRS group might be linked to signal transduction and structural components of epidermis under chronic stress (Lee, 2011). It is also reported that lysophosphatidylcholine (LPC) levels in the prefrontal cortex in brain are directly correlated with blood corticosterone levels (Oliveira et al., 2016). Another research shows that excessive expression of LPCs is correlated with high oxidative stress (Hung et al., 2020). Typically, stress is characterized by activation of the sympathetic nervous system and hypothalamic–pituitary–adrenal axis, resulting in release of glucocorticoids (Marin et al., 2007). Chronic stress increases the levels of corticosterone to extend HFSC quiescence and inhibit hair growth in mice, which indicates that LPC may be related to hair growth under stress (Choi et al., 2021).

Lipid metabolism is thought to play an essential role in maintaining normal physiological cellular functions and involving in hair development and function (W. S. Lee, 2011; Palmer et al., 2020). Thus, our current investigation took advantage of metabolomics and transcriptomics analysis to further verify the metabolomics results and showed mRNA levels of the relevant glycerophospholipid metabolism were significantly increased, such as *Agpat2, Gpd1, Gapt3, Chpt1, and Lpin1;* while others such as *plb1, Pla2g2e, Pla2g2d, and Lpin3* were decreased. The expression of *Lpl* involved in the glycerolipid metabolism was also significantly increased. Changes in these genes might suggest the underlying connection between CRS inhibit hair growth and lipid metabolism. We also found that genes related to ATP-binding cassette (ABC) transporter, such as *Abca8a, Abca9, Abcd2, Abca8b, and Abcc6* are all significantly up-regulated. ABC transporters mediate the transport of lipids. In particular, the ABCA family is involved in both cholesterol efflux and intracellular transport (Quazi and Molday, 2011; Tarling et al., 2013). It has been revealed that cholesterol modulates HF cycling by regulating bone morphogenic protein (BMP) family members, Wnt/β-catenin and Notch pathways (Cooper et al., 2003; Lee and Tumbar, 2012; Mathay et al., 2011; Sheng et al., 2014), which indicates the importance of cholesterol homeostasis in stress inhibit hair growth.

Other metabolic pathways involved in carbohydrate metabolism (fructose and mannose metabolism, galactose metabolism, amino sugar and nucleotide sugar metabolism, starch and sucrose metabolism, pentose phosphate pathway, pentose and glucuronate interconversions) are also changed significantly after CRS treatment during the hair growth. Compared to control group, expression of Hk-1 decreased markedly in skin tissues of CRS group in all 5 primary metabolic pathways. Hk-1 encodes a ubiquitous form of hexokinase which localizes on the outer membrane of mitochondria. Hexokinases catalyzes the conversion of glucose to glucose-6-phosphate in the first step of glycolytic metabolism. Then glucose-6-phosphate convert to fructose-6-phosphate catalyzed by glucose-6-phosphate isomerase (GPI) in glycolysis. As mentioned above, D-glucose 6-phosphate was the most significantly down-regulated metabolite in secondary metabolites. This is usually attributed to Hk-1 activity decrease or to G6P dehydrogenase (G6PD) activity increase (Rodriguez-Rodriguez et al., 2013). G6PD catalyzes the oxidation of glucose-6-phosphate to 6-phosphogluconate (Kotaka et al., 2005). This transformation is a rate-limiting step of the pentose-phosphate pathway, which represents a route for the dissimilation of carbohydrates besides glycolysis (Kirsch et al., 2009). However, the two genes we identified relating to G6P Dehydrogenase, *G6pdx and G6pd2* exhibited no significant difference between CRS group and control group. Thus, we presume that Hk-1 may contribute to the decrease of D-glucose 6-phosphate, which was another piece of evidence proving the disturbances in the glycolytic metabolism. However, the function of Hk-1 in CRS induced hair growth inhibition is still unknown. Therefore, we will assess the hexokinase activity and construct the conditional knockout Hk-1 mice in hair follicle stem cell to research the mechanisms and function of Hk-1 *in vivo* and *vitro*.

However, most metabolites in TCA cycle were not significantly changed between the CRS group and control group, which suggested that glycolytic metabolism rather than TCA cycle might play an important role in hair growth under CRS. It has been reported that HFSC likely have relatively higher levels of glycolysis compared to the rest of the epidermis, which indicates the changes of glycolysis metabolite may be linked to metabolic status of HFSC(Flores et al., 2017). HFSCs quickly in response to the barrage of cues which is dependent on increasing glycolytic rate that orchestrates the onset of a new hair cycle, whereas chronic stress may prolong HFSC quiescence and maintain hair follicles in an extended resting phase (Choi et al., 2021). Glutamine metabolism also regulates hair follicle stem cell progenitor state (C. S. Kim et al., 2020). In our study, we found the metabolic features such as glutamine and glutamate were downregulated in skin tissues of CRS group. The rapidly proliferating stem cells required ATP as well as nucleotides, aerobic glycolysis, which could also explain the alter of genes involved in purine metabolism (Ahmed et al., 2018). Although, there are numerous researchers take advantage of mouse model to explore the mechanisms of hair loss and the regulation of hair follicle cycling. For example, it has been reported that JAK inhibition regulates the activation of key hair follicle populations to promote the hair growth in both mouse and human by topical treatment (Harel et al., 2015). Furthermore, there has been reported that the retinoid metabolism is altered in human and mouse cicatricial alopecia (Everts et al., 2013). However, the function of these pathways still needs to be further investigated and the differences between the model of murine hair growth and human scalp hair growth should be concerned. Moreover, we will further explore the metabolism changes in hair-loss patients caused by stress and compare with the mouse model to find the similar metabolic changes.

## Conclusion

In this study, we discovered that CRS suppressed hair growth, found the metabolism pathways including carbohydrate metabolism, amino acid metabolism, lipid metabolism were significantly changed, and revealed the metabolism associated DEGs such as Hk-1 by transcriptomics and metabolomics analyses in skin tissues of C57BL/6 mice. Our results provided new insights into the molecular mechanisms of CRS-induced hair growth inhibition and indicated that targeting to specific metabolic pathways might be useful for therapy of CRS inhibit hair growth.

## Data Availability

Based on our identifications, metabolite information was submitted to MetaboLights public repository (www.ebi.ac.uk/metabolights/MTBLS4085). The transcriptomics data presented in the study are deposited in the Sequence Read Archive repository, accession number (PRJNA763049). The datasets can be found in online repositories (https://www.ncbi.nlm.nih.gov/sra/?term=PRJNA763049).
