# Supplementary material for: Integrated Transcriptomics and Metabolomics Analyses of Stress-Induced Murine Hair Follicle Growth Inhibition
Source: Front Mol Biosci. 2022 Feb 7;9:781619. doi: 10.3389/fmolb.2022.781619 (PMC8859263; doi:10.3389/fmolb.2022.781619)
Supplement: Supplementary file 2 [file Table3.docx]

**Supplementary Tables**

**Supplementary Table 3. The FPKM values of significantly differentiated genes associated with primary metabolites.**

| gene_  name | FPKM.CRS_1 | FPKM.CRS_2 | FPKM.CRS_3 | FPKM.Ctrl_1 | FPKM.Ctrl_2 | FPKM.Ctrl_3 |
| --- | --- | --- | --- | --- | --- | --- |
| Nanp | 0.00 | 0.00 | 0.00 | 3.70 | 4.04 | 2.96 |
| Cmah | 1.21 | 1.06 | 2.37 | 10.22 | 9.37 | 10.14 |
| Hk1 | 1.96 | 1.62 | 1.70 | 3.40 | 4.23 | 3.97 |
| Cyb5r2 | 1.59 | 1.19 | 0.93 | 0.63 | 0.61 | 0.33 |
| Agl | 4.82 | 7.48 | 4.91 | 2.36 | 2.75 | 2.92 |
| Gbe1 | 6.20 | 10.34 | 5.18 | 2.91 | 3.42 | 3.74 |
| Amy1 | 3.19 | 4.71 | 3.33 | 1.19 | 2.68 | 1.16 |

**Supplementary Table 4. The FPKM values of significantly differentiated genes associated with secondary metabolites.**

| gene_  name | | FPKM.CRS_1 | FPKM.CRS_2 | | FPKM.CRS_3 | FPKM.Ctrl_1 | | FPKM.Ctrl_2 | FPKM.Ctrl_3 | |
| --- | --- | --- | --- | --- | --- | --- | --- | --- | --- | --- |
| Agpat2 | 31.04 | | | 43.39 | 38.48 | 11.16 | 6.96 | | 11.28 |  |
| Plb1 | 0.30 | | | 0.22 | 0.37 | 1.01 | 1.44 | | 1.06 |  |
| Lpin3 | 8.38 | | | 6.10 | 12.86 | 23.57 | 19.26 | | 24.49 |  |
| Lpin1 | 5.70 | | | 5.44 | 4.82 | 2.22 | 2.55 | | 2.59 |  |
| Gpd1 | 67.39 | | | 101.02 | 75.03 | 25.88 | 48.09 | | 25.88 |  |
| Pla2g2e | 5.30 | | | 1.83 | 6.28 | 17.56 | 17.30 | | 14.60 |  |
| Chpt1 | 5.55 | | | 8.38 | 5.59 | 1.80 | 3.29 | | 2.86 |  |
| Pla2g2d | 0.60 | | | 0.71 | 0.41 | 1.74 | 1.27 | | 1.29 |  |
| Gpat3 | 3.34 | | | 4.88 | 3.80 | 1.41 | 2.72 | | 1.53 |  |
| Lpl | 61.78 | | | 83.78 | 67.00 | 25.35 | 53.61 | | 24.18 |  |

**Supplementary Table 5. The FPKM values of significantly differentiated genes in arachidonic acid metabolism**

| gene_  name | FPKM.CRS_1 | FPKM.CRS_2 | FPKM.CRS_3 | FPKM.Ctrl_1 | FPKM.Ctrl_2 | FPKM.Ctrl_3 |
| --- | --- | --- | --- | --- | --- | --- |
| Ptgds | 33.03 | 24.11 | 46.09 | 102.22 | 87.02 | 84.83 |
| Gpx7 | 19.93 | 19.59 | 16.51 | 5.56 | 6.02 | 5.06 |
| Plb1 | 0.30 | 0.22 | 0.37 | 1.01 | 1.44 | 1.06 |
| Cyp2b19 | 17.88 | 17.03 | 18.74 | 37.30 | 36.85 | 35.29 |
| Gpx3 | 184.41 | 209.62 | 128.59 | 59.81 | 90.46 | 61.72 |
| Ggt1 | 12.89 | 8.97 | 15.05 | 26.15 | 26.57 | 24.20 |
| Pla2g2e | 5.30 | 1.83 | 6.28 | 17.56 | 17.30 | 14.60 |
| Alox12 | 2.02 | 1.46 | 1.28 | 4.62 | 4.00 | 4.28 |
| Cyp2e1 | 19.40 | 46.31 | 22.62 | 1.90 | 11.74 | 2.11 |
| Ptges | 9.80 | 10.24 | 6.68 | 3.69 | 4.73 | 4.51 |
| Pla2g2d | 0.60 | 0.71 | 0.41 | 1.74 | 1.27 | 1.29 |
| Ptgis | 0.68 | 1.47 | 0.42 | 0.16 | 0.33 | 0.41 |

**Supplementary Table 6. The FPKM values of significantly differentiated genes in glutathione metabolism**

| gene_  name | FPKM.CRS_1 | FPKM.CRS_2 | FPKM.CRS_3 | FPKM.Ctrl_1 | FPKM.Ctrl_2 | FPKM.Ctrl_3 |
| --- | --- | --- | --- | --- | --- | --- |
| Chac1 | 8.89 | 7.47 | 10.47 | 46.90 | 36.46 | 42.28 |
| Oplah | 11.90 | 11.86 | 16.06 | 27.94 | 30.99 | 32.12 |
| Gpx7 | 19.93 | 19.59 | 16.51 | 5.56 | 6.02 | 5.06 |
| Gpx3 | 184.41 | 209.62 | 128.59 | 59.81 | 90.46 | 61.72 |
| Ggt1 | 12.89 | 8.97 | 15.05 | 26.15 | 26.57 | 24.20 |
| Gsta3 | 1.49 | 2.82 | 1.27 | 0.30 | 0.85 | 0.31 |

**Supplementary Table 7. The FPKM values of significantly differentiated genes in glycolysis gluconeogenesis**

| gene_  name | FPKM.CRS_1 | FPKM.CRS_2 | FPKM.CRS_3 | FPKM.Ctrl_1 | FPKM.Ctrl_2 | FPKM.Ctrl_3 |
| --- | --- | --- | --- | --- | --- | --- |
| Hk1 | 1.96 | 1.62 | 1.70 | 3.40 | 4.23 | 3.97 |
| Aldh1b1 | 1.27 | 0.90 | 1.61 | 2.87 | 2.80 | 2.10 |
| Adh1 | 15.78 | 19.10 | 11.45 | 4.22 | 6.92 | 5.45 |
| Aldh3a1 | 18.93 | 16.59 | 9.40 | 3.41 | 8.70 | 7.23 |

**Supplementary Table 8. The FPKM values of significantly differentiated genes in nicotinate and nicotinamide metabolism**

| gene_  name | FPKM.CRS_1 | FPKM.CRS_2 | FPKM.CRS_3 | FPKM.Ctrl_1 | FPKM.Ctrl_2 | FPKM.Ctrl_3 |
| --- | --- | --- | --- | --- | --- | --- |
| Aox4 | 3.83 | 2.66 | 3.02 | 1.50 | 1.86 | 1.33 |
| Nnt | 0.27 | 0.19 | 0.45 | 1.90 | 0.69 | 0.51 |
| Aox1 | 2.29 | 4.44 | 2.26 | 0.81 | 1.54 | 1.39 |
| Aox3 | 0.51 | 0.56 | 0.37 | 0.25 | 0.21 | 0.10 |
| Nnmt | 9.14 | 12.18 | 9.32 | 3.40 | 7.58 | 3.49 |
| Nmnat2 | 1.04 | 1.84 | 4.06 | 1.26 | 0.73 | 1.18 |
| Bst1 | 0.63 | 0.45 | 0.39 | 0.25 | 0.25 | 0.23 |
| Qprt | 0.36 | 0.38 | 0.05 | 0.02 | 0.17 | 0.08 |

**Supplementary Table 9. The FPKM values of significantly differentiated genes in purine metabolism**

| gene_  name | FPKM.CRS_1 | FPKM.CRS_2 | FPKM.CRS_3 | FPKM.Ctrl_1 | FPKM.Ctrl_2 | FPKM.Ctrl_3 |
| --- | --- | --- | --- | --- | --- | --- |
| Gucy2c | 0.08 | 0.07 | 0.12 | 0.41 | 0.51 | 0.47 |
| Entpd8 | 0.01 | 0.00 | 0.00 | 0.43 | 0.49 | 0.61 |
| Adcy7 | 1.05 | 1.01 | 4.21 | 0.77 | 1.38 | 0.92 |
| Pde1a | 0.57 | 0.71 | 0.42 | 0.13 | 0.32 | 0.13 |
| Pde3b | 1.16 | 1.73 | 1.35 | 0.36 | 0.83 | 0.31 |
| Pde3a | 0.97 | 0.90 | 1.00 | 0.43 | 0.36 | 0.46 |
| Gucy1b1 | 1.13 | 1.26 | 1.38 | 0.40 | 0.77 | 0.39 |
| Gucy1a1 | 0.81 | 1.04 | 0.82 | 0.29 | 0.61 | 0.36 |
| Pde2a | 0.93 | 0.22 | 2.12 | 0.04 | 0.07 | 0.60 |
| Pde4d | 1.46 | 1.90 | 1.50 | 0.26 | 0.67 | 0.55 |
| Pde10a | 0.34 | 0.33 | 0.20 | 0.17 | 0.11 | 0.13 |
| Pde7b | 0.49 | 0.74 | 0.57 | 0.27 | 0.39 | 0.21 |

**Supplementary Table 10. The FPKM values of significantly differentiated genes in retinol metabolism**

| gene_  name | FPKM.CRS_1 | FPKM.CRS_2 | FPKM.CRS_3 | FPKM.Ctrl_1 | FPKM.Ctrl_2 | FPKM.Ctrl_3 |
| --- | --- | --- | --- | --- | --- | --- |
| Cyp2b19 | 17.88 | 17.03 | 18.74 | 37.30 | 36.85 | 35.29 |
| Aldh1a1 | 19.76 | 27.46 | 15.70 | 7.80 | 9.94 | 9.43 |
| Adh1 | 15.78 | 19.10 | 11.45 | 4.22 | 6.92 | 5.45 |
| Dhrs9 | 1.39 | 0.83 | 1.78 | 3.21 | 3.58 | 3.67 |
| Aox4 | 3.83 | 2.66 | 3.02 | 1.50 | 1.86 | 1.33 |
| Aox1 | 2.29 | 4.44 | 2.26 | 0.81 | 1.54 | 1.39 |
| Aldh1a7 | 4.68 | 7.97 | 5.45 | 0.77 | 3.64 | 1.58 |
| Cyp1a1 | 1.87 | 1.17 | 0.89 | 0.42 | 0.52 | 0.67 |
| Aox3 | 0.51 | 0.56 | 0.37 | 0.25 | 0.21 | 0.10 |
| Bco1 | 1.09 | 1.06 | 0.76 | 1.79 | 2.34 | 1.95 |

**Supplementary Table 11. The FPKM values of significantly differentiated genes in ABC transporters**

| gene_  name | FPKM.CRS_1 | FPKM.CRS_2 | FPKM.CRS_3 | FPKM.Ctrl_1 | FPKM.Ctrl_2 | FPKM.Ctrl_3 |
| --- | --- | --- | --- | --- | --- | --- |
| Abca8a | 4.87 | 8.87 | 3.78 | 1.84 | 2.61 | 1.85 |
| Abca9 | 1.88 | 2.24 | 1.54 | 0.81 | 0.78 | 0.62 |
| Abcd2 | 5.23 | 6.99 | 5.21 | 1.32 | 4.15 | 1.64 |
| Abca8b | 0.91 | 1.53 | 0.73 | 0.36 | 0.51 | 0.41 |
| Abcc6 | 0.31 | 0.38 | 0.17 | 0.07 | 0.11 | 0.16 |
