# Supplementary material for: Integrated Transcriptomics and Metabolomics Analyses of Stress-Induced Murine Hair Follicle Growth Inhibition
Source: Front Mol Biosci. 2022 Feb 7;9:781619. doi: 10.3389/fmolb.2022.781619 (PMC8859263; doi:10.3389/fmolb.2022.781619)
Supplement: Supplementary file 4 [file DataSheet1.docx]

Supplementary Figures


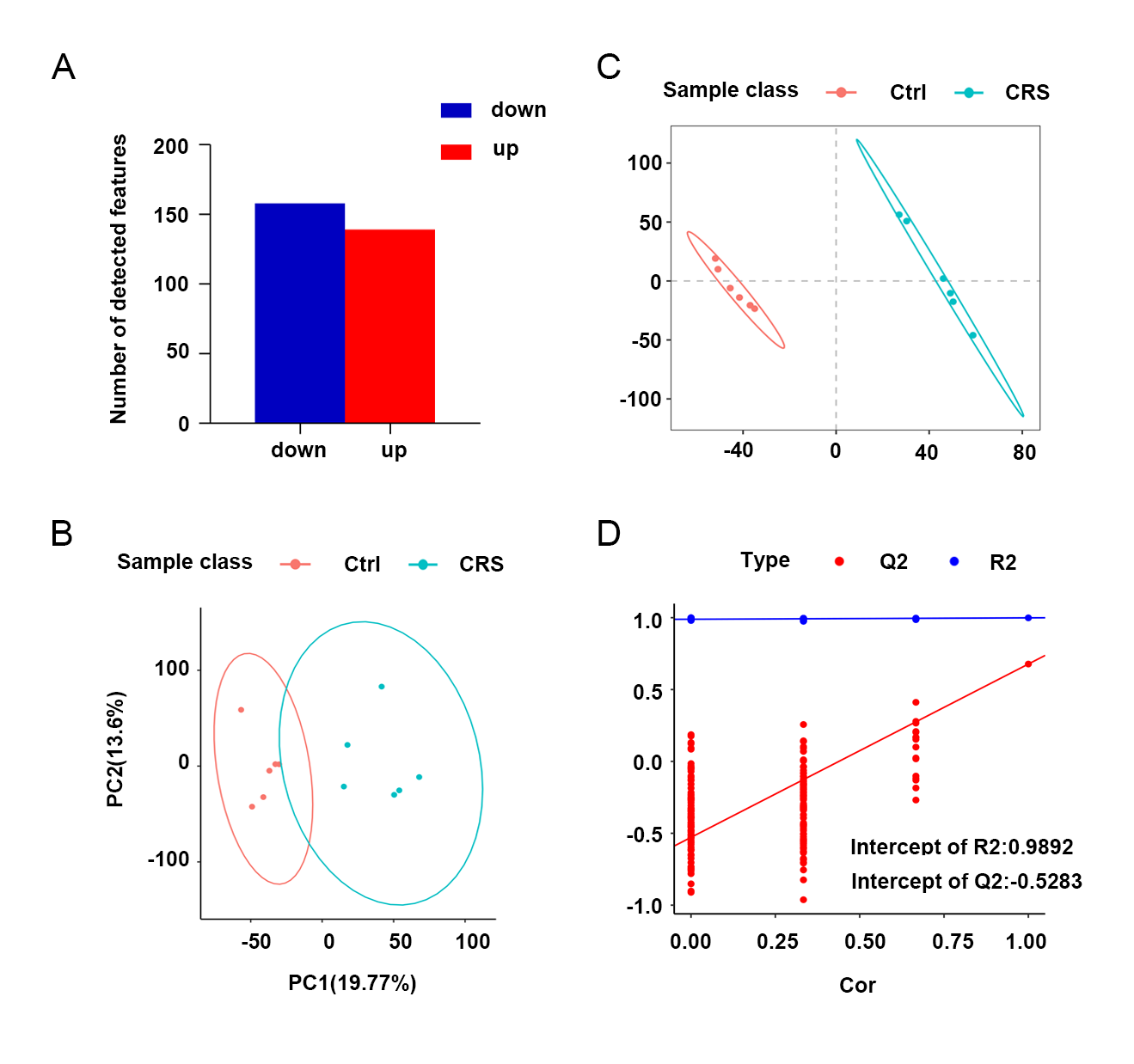


**Supplementary Figure 1. CRS significantly regulates metabolic profile of the skin tissue**

1. Numbers of significantly differential metabolites detected between CRS group and control group.
2. PCA based on metabolite analysis in skin tissues of CRS group and control group (combined positive and negative ion modes).
3. PLS- DA score graph of comparison between CRS and control group.
4. PLS- DA displacement test results of comparison between CRS and control group.


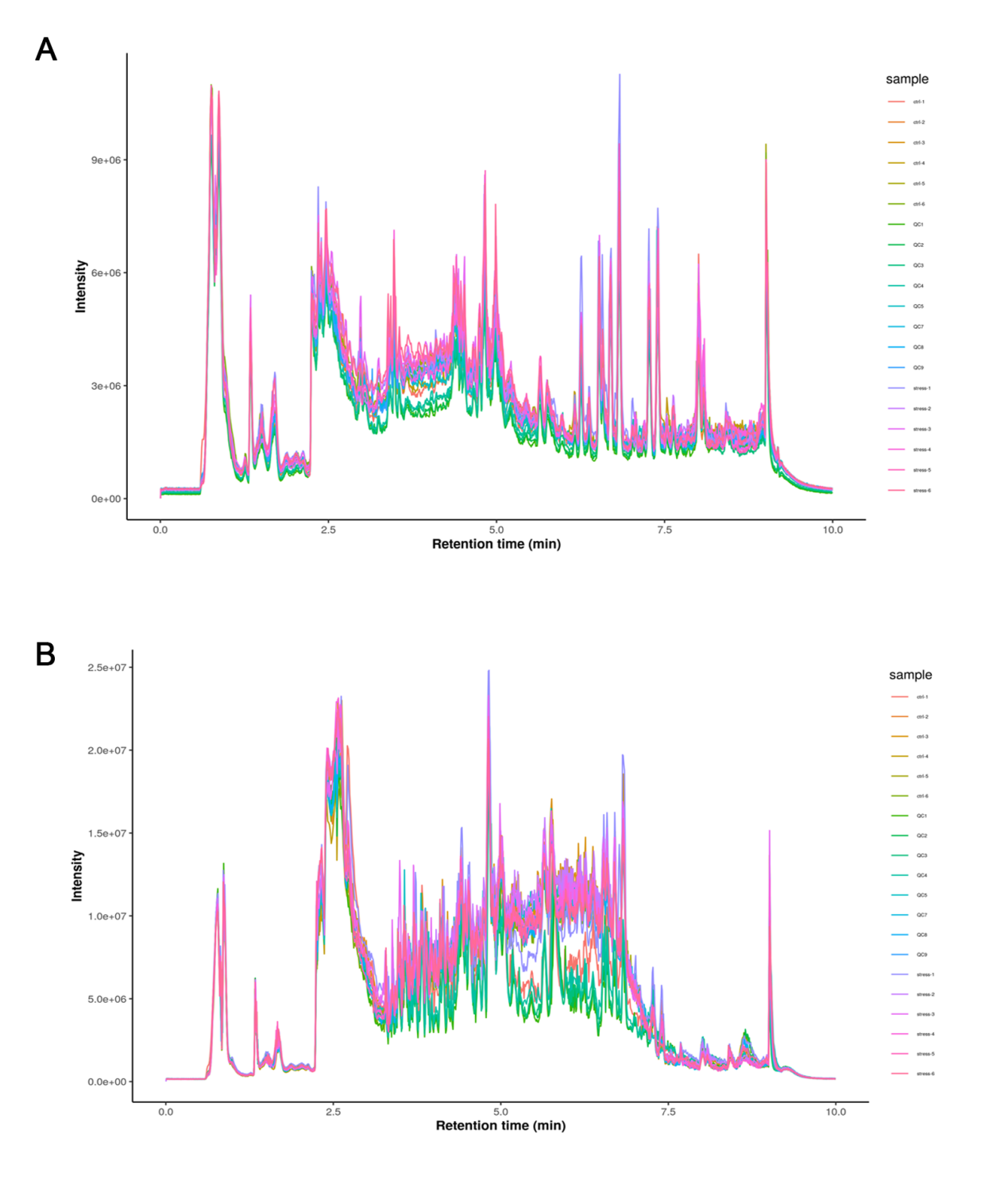


**Supplementary Figure 2. TIC chromatograms and retention time width of metabolites in the dorsal skin of mice in CRS and control group.**

1. TIC chromatograms and retention time width of metabolites in the dorsal skin of mice in negative mode.
2. TIC chromatograms and retention time width of metabolites in the dorsal skin of mice in positive mode.


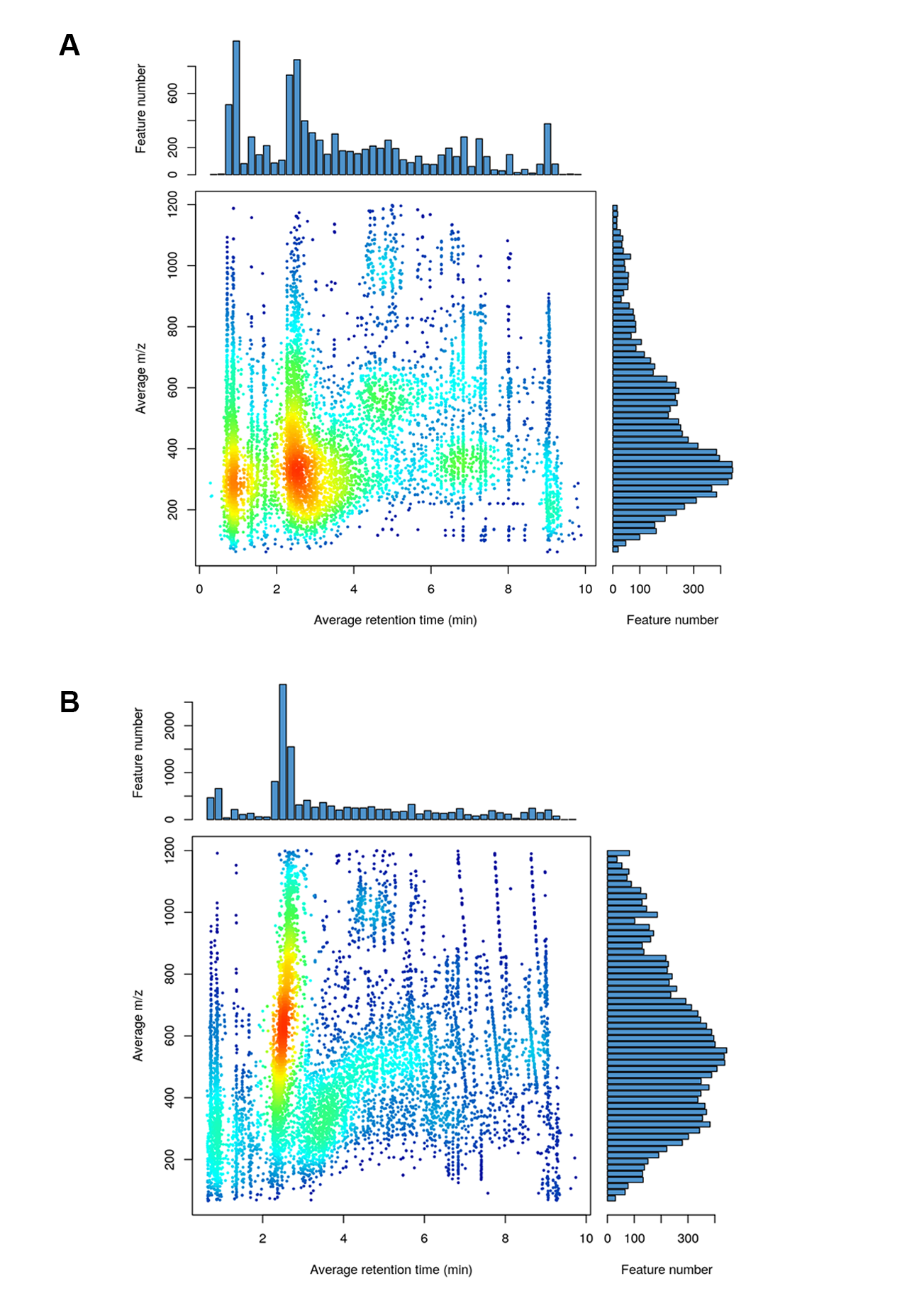


**Supplementary Figure 3. Metabolite m/z -RT distribution in the dorsal skin of mice in CRS and control group.**

1. Analysis of average m/z distribution, metabolite intensity distribution and coefficient of variation distribution detected by negative ion mode.
2. Analysis of average m/z distribution, metabolite intensity distribution and coefficient of variation distribution detected by positive ion mode.


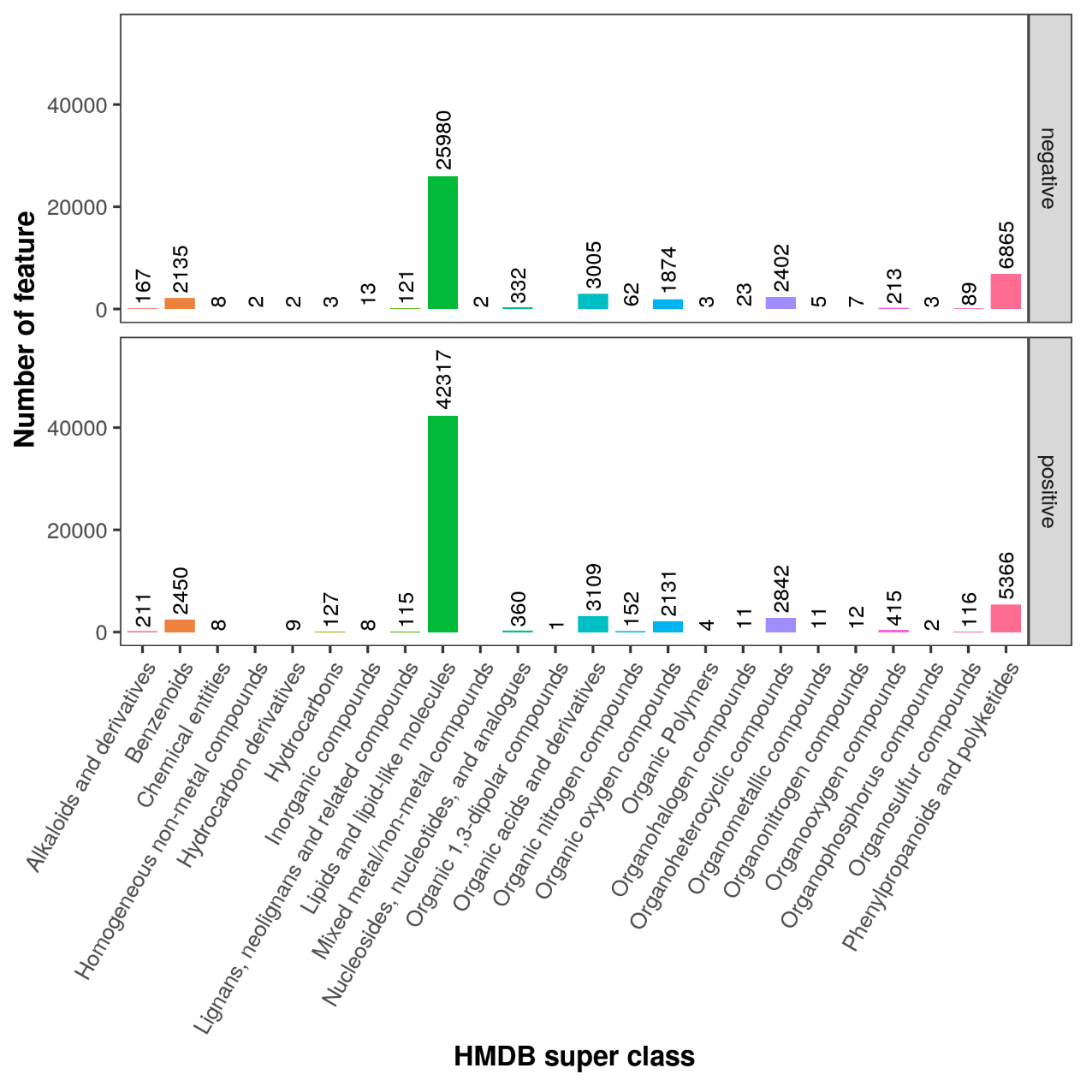


**Supplementary Figure 4. Human Metabolome Database (HMDB) classification map in positive ion mode and negative ion mode.**

**
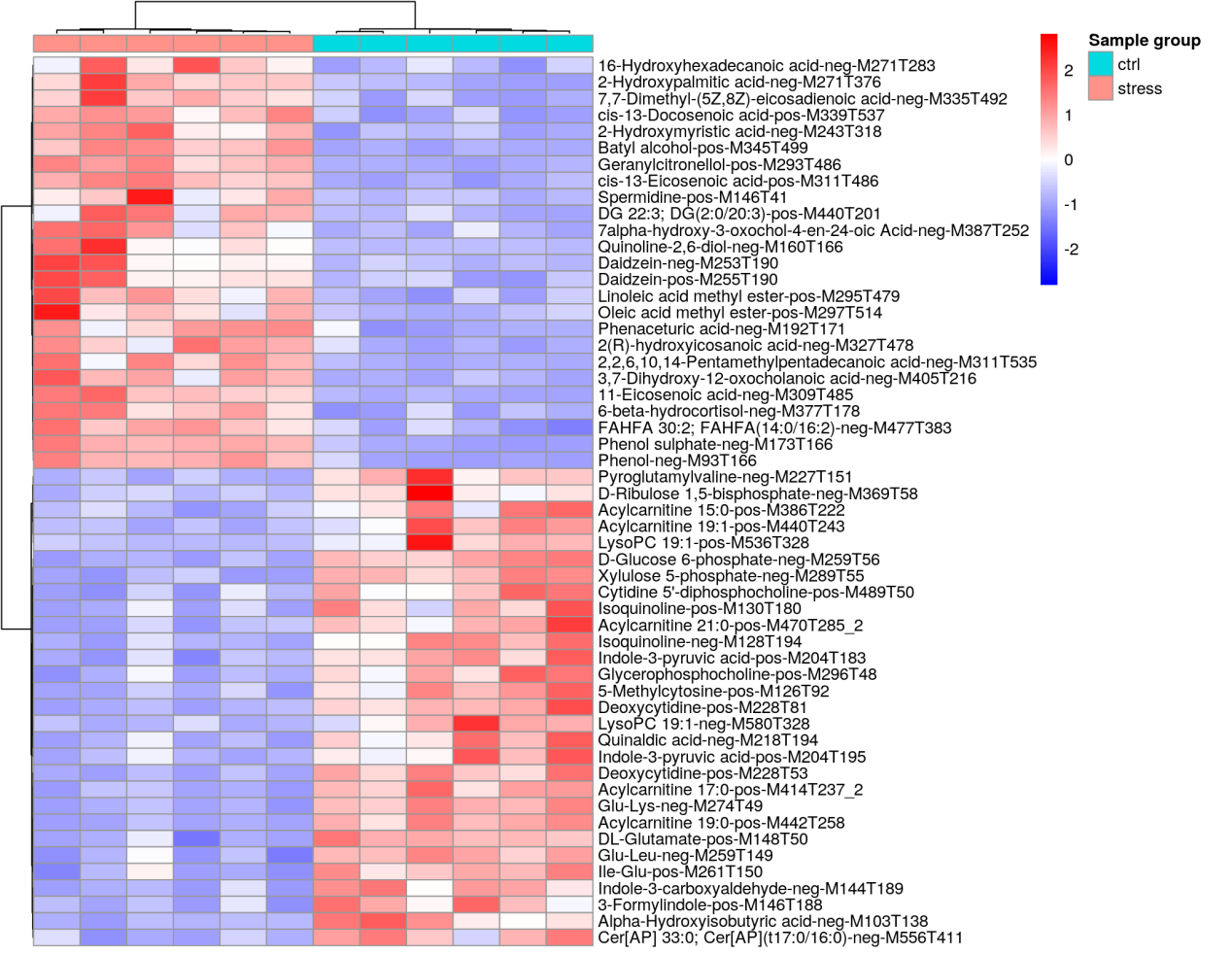
**

**Supplementary Figure 5. Heatmap analysis showed differential secondary metabolites.**


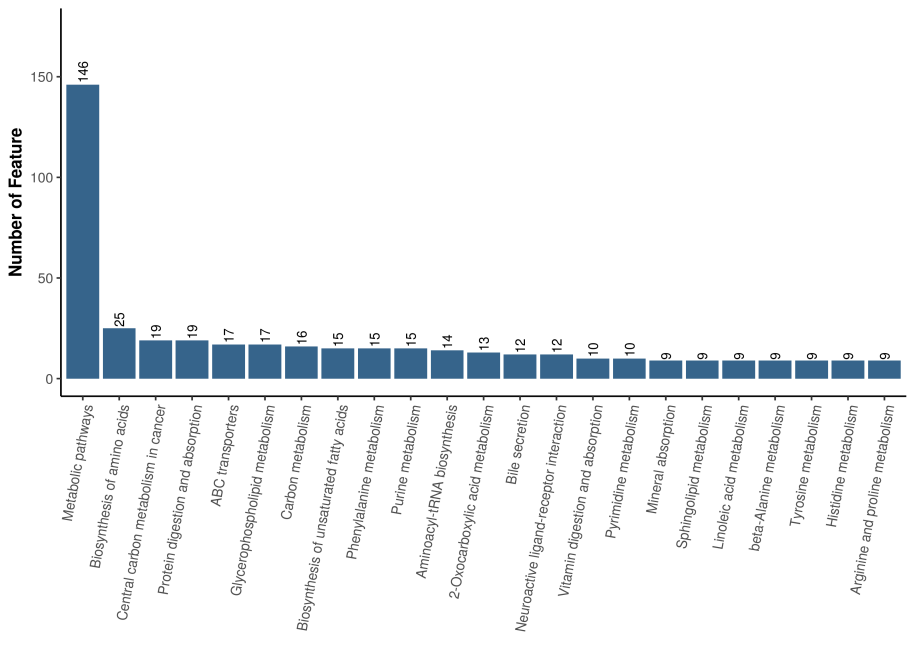


**Supplementary Figure 6. Top 20 KEGG pathways of secondary metabolites.**


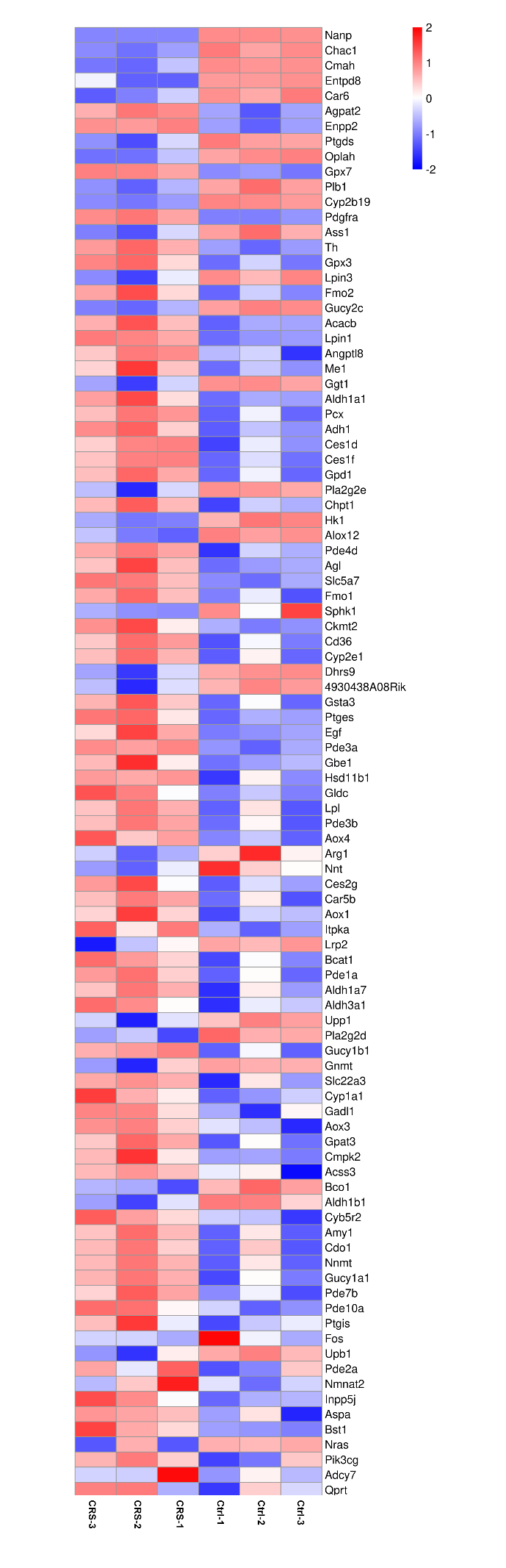


**Supplementary Figure 7. CRS significantly alters genes expression related to metabolism pathways based on RNA-seq.**

Heatmap analysis showed significantly DEGs associated with metabolism.
